# Experiences Reported by People with Epilepsy During Antiseizure Medication Shortages in the UK: A Cross-Sectional Survey

**DOI:** 10.3390/pharmacy13060166

**Published:** 2025-11-10

**Authors:** Eric Amankona Abrefa Kyeremaa, Tom Shillito, Caroline Smith, Charlotte Lawthom, Sion Scott, David Wright

**Affiliations:** 1 School of Healthcare, University of Leicester, Leicester LE1 7RH, UK; 2 Epilepsy Action, Leeds LS19 7XY, UK; 3 Aneurin Bevan University Health Board, Newport NP11 5GH, UK

**Keywords:** out of stock, epilepsy medicine, ASM, quantitative study, brand switching, community pharmacy service

## Abstract

(1) Background: Medication shortages have become increasingly common in the UK. However, there is limited evidence regarding the experiences of people with epilepsy and their caregivers during these shortages. The aim of this study is to explore the extent and impact of ASM shortages on people with epilepsy and their caregivers across the UK. (2) Methods: A cross-sectional online survey was distributed between January and April, 2024 by epilepsy charities. Participants included people with epilepsy and caregivers. The survey collected demographic information, types of ASM respondents were prescribed, experiences of shortages, and the impact of shortages. Data were analysed descriptively, and subgroup analyses were conducted by medication type. (3) Results: A total of 1549 responded, of whom 1312 were people with epilepsy and their carers who were included in the analysis with a mean age of 43 years. A total of 941 respondents (71.7%) reported difficulty obtaining their prescribed ASM in the past year. Shortages were most frequently reported for sodium valproate (60.8%), lamotrigine (65.2%), carbamazepine (92.6%), clobazam (82.6%), topiramate (81.5%), zonisamide (74.0%), levetiracetam (62.8%), lacosamide (71.0%), and brivaracetam (70.5%). A total of 529 (40.4%) of the participants reported that stress and/or anxiety caused by medication shortages was associated with recurrent seizures. We did not ask whether patients missed medications because of these difficulties. (4) Conclusions: ASM shortages are a widespread issue for people with epilepsy in the UK, leading to treatment disruptions and psychological distress. Addressing supply change limitations and identifying effective approaches to preventing the substitution of ASMs brands by clinicians may potentially reduce this problem.

## 1. Introduction

Epilepsy is the most common neurological condition worldwide, affecting approximately 50 million people globally [1,2]. In the United Kingdom (UK), an estimated 600 people are newly diagnosed with the condition each week [3]. The condition is characterised by recurrent, unprovoked seizures, which can vary in type and severity and often have a substantial effect on daily life [4]. In addition to the physical impact of epilepsy, the psychological impact is also reported to be significant, including anxiety, stress, and depression [2,5]. In Western healthcare systems, the primary treatment for epilepsy is with antiseizure medication (ASM). These medicines are effective in controlling seizures in approximately 7 out of 10 people with epilepsy [6].

Despite the benefits of ASM, international shortages of these medications have been regularly reported [7,8,9,10]. Typically, the supply chain of pharmaceutical drugs involves the supply of raw materials to the manufacturer, the manufacturing of the medicine, transfer to the wholesaler, and finally distribution to the pharmacy where the medication is dispensed to patients [7].

Shortages may arise due to several factors, including a lack of raw materials, the medicine being out of stock at the wholesale level due to manufacturing problems, or the medicine not being available at a specific pharmacy.

In the UK, the National Health Service (NHS) has faced numerous drug shortages, with the number of supply disruptions doubling between 2022 and 2024, rising from 52 in February 2022 to 101 in February 2024, causing serious impacts on both patients and healthcare providers [11,12,13]. When a pharmacy is unable to obtain a medicine, patients are sent back to their primary care prescriber for their prescription to be changed to an alternative medicine. This places patients at a disadvantage as they are not able to obtain their medicines when they need them and their alternative medicine may not be appropriate for them [14].

Shortages of medicines in the UK have been linked to disruptions in supply chains triggered by events such as the COVID-19 pandemic and the Ukraine war causing rises in energy costs, which has affected the manufacturing and transport of essential pharmaceutical materials [15]. Post the United Kingdom leaving the European Union, new regulations have resulted in border delays and customs checks, which, coupled with the depreciation of the currency, have made medicine importation difficult and expensive [16].

Issues with medication supply may have a particular impact on people with epilepsy, as many need to remain on the same brand of medicine due to differences in bioequivalence resulting in changes in seizure control or side effects [17]. Epilepsy medicines are frequently classified by whether they can be safely swapped for another product. In the UK, the Medicines and Healthcare Regulatory Agency classifies ASM as Category 1, switching and substitution should be avoided as equivalence cannot be assumed; Category 2, switching should be performed with care; and Category 3, therapeutic equivalence can be assumed between different brands and generics. However patient factors such as anxiety, risk of confusion, or potential for dosing errors should be taken into account when switching between brands [17].

It is therefore more difficult for people with epilepsy to switch product if a manufacturing shortage occurs, wholesalers choose not to stock certain products, or community pharmacies limit themselves to a small number of wholesalers for supplies.

People with epilepsy have reported that changes to their medication as a result of AMS shortages can lead to increased side effects and seizure frequency [18]. Shortages can additionally impact them psychologically, causing fear and anxiety, as well as frustration. Furthermore, shortages can lead to missed doses, which can increase seizure frequency and/or severity, disrupt sleep patterns, work schedules, and social activities, and affect epilepsy care [18].

Following several reports of ASM shortages through UK epilepsy charity helplines, it was decided to survey people with epilepsy to ascertain the extent of the problem and its potential impact [19].

Aims and Objectives

The aim of this study is to

1. Describe the frequency of ASM shortages in the UK;
2. Compare reported shortages with available national database recording of pharmaceutical supply disruptions to reported shortages to identify where problems in the supply chain may be occurring;
3. Identify the difficulties associated with accessing prescribed ASM and switching between brands;
4. Evaluate the quality of support provided by pharmacists;
5. Explore the impact of shortages on respondents.

## 2. Materials and Methods

### 2.1. Ethical Approval

According to the Health Research Authority (HRA) decision tool (2025), this study falls under the category of service evaluation and meets the conditions for exemption from formal ethical review [20]. The survey was sent to members of the different charities who had previously agreed to receive surveys of this nature, and therefore the charitable bodies did not seek external ethical approval. Data was provided anonymously to the academic partners for analysis.

### 2.2. Study Design and Period

A cross-sectional study design was employed to explore access to ASMs and the impact of medication shortages among individuals with epilepsy in the United Kingdom. This study followed the Consensus-Based Checklist for Reporting of Survey Studies. The study was conducted between 6 January 2024 and 1 April 2024, and data were collected via the Survey Monkey online platform (SurveyMonkey Inc., San Mateo, CA, USA). Participants were recruited using purposive convenience sampling as this approach allowed for the recruitment of individuals who were readily accessible and experiencing a medication shortage.

### 2.3. Study Population

The participants of this study were recruited from members of Epilepsy Action, Epilepsy Society, and SUDEP Action, which are UK-based epilepsy charities, and Parkinson’s UK, a UK-based charity supporting people with Parkinson’s disease. The survey was sent to 56,000 people with epilepsy and Parkinson’s and their caregivers. The majority of survey respondents were service users of Epilepsy Action, of whom approximately 17% were male, 35% were female, 1% identified as ‘other’, and the remaining 47% preferred not to say their gender.

### 2.4. Inclusion Criteria

- A service user, member, or supporter of Epilepsy Action, Epilepsy Society, SUDEP Action, or Parkinson’s UK;
- Residing within the UK;
- Individual with a clinical diagnosis of epilepsy or caring for someone with epilepsy.

### 2.5. Recruitment Process

The participants were invited to participate in this study through advertisements in the charity’s newsletters, social media platforms (X and Facebook), and mailing lists. The advertisement included the following:

- The study aim;
- What the data would be used for;
- The survey closing date;
- Logos of the four charities involved.

### 2.6. Survey Content

Data were collected using a structured questionnaire that consisted of 26 items. The questionnaire included sections on demographic information, access to antiseizure medication, and the impact of medication shortages in the UK. The questions were simple in format, consisting of dichotomous (yes/no), Likert scale items, and open-ended questions. See Supplementary Files S1–S6 for the questionnaire used. Demographic data collected included

- Gender;
- Age;
- Medication prescribed;
- Method of collecting prescription.

The questionnaire was developed collaboratively between the four charities and patient representatives through a series of meetings. The final version was piloted with service users of Epilepsy Action to assess the clarity of the questions. Feedback resulted in revisions to simplify language and improve question flow.

### 2.7. Improving Response Rate

In line with Cochrane Review evidence [21], the following elements were included within the survey development process to increase the probability of responses:

- A topic of interest: the survey focused on ASM shortages, following multiple reports to UK epilepsy charity helplines.
- Textual presentation for responses: questions were presented in a clear, text-based format.
- Follow up reminder: there were monthly reminders in charities newsletter during the duration of the data collection.
- Use of charity logo: the logos of all participating charities were displayed on the questionnaire to reassure respondents of the study’s credibility and legitimacy.
- Did not mention ‘survey’ in the subject line: The word “survey” was avoided in subject lines to reduce the risk of emails being ignored or filtered as spam.
- A statement such as complete this “brief survey” was added to the advertisement message.
- Personalised email: where possible, invitations were personalised (e.g., using the recipient’s name), which is known to increase engagement.
- Inclusion of a picture: a relevant image was added to capture attention and make the invitation visually engaging.
- The information about the potential benefits of the study was shared with members of the charity with the aim of improving the response rate.

### 2.8. Data Processing and Analysis

The data from this study is a subset of a larger database that includes people with epilepsy and/or Parkinson’s and their caregivers. Only respondents who self-reported a diagnosis of epilepsy or identified themselves as carers of someone with epilepsy were included in the analysis. Responses were exported into Microsoft Excel and analysed using IBM SPSS Statistics version 29. Descriptive statistics (frequencies and percentages) were used to summarise participant characteristics and responses. Manufacturer reports of shortages were obtained from the Epilepsy Action Drugwatch archives webpage [22] and compared to those provided by survey respondents.

Chi-squared tests were conducted to explore associations between difficulty accessing ASM and specific medication prescribed (carbamazepine, sodium valproate, lamotrigine, clobazam, topiramate, zonisamide, levetiracetam, lacosamide, brivaracetam). Incomplete survey responses were included in the data analysis process.

Open-ended responses were analysed using content analysis [23] to identify common topics related to ASM shortages in the UK and describe the quality of support provided by pharmacists and the impact of shortages. This involved reviewing narrative responses, coding recurring patterns, and extracting representative quotations to illustrate findings using NVivo software 14. The coding was initially performed by EK and reviewed independently by DW to ensure consistency in interpretation.

## 3. Results

### 3.1. Demographics

Participant Characteristics

A total of 1549 responses were received for this study, including individuals with epilepsy or Parkinson’s disease and their caregivers. Of these, 1312 responses from people with epilepsy and their caregivers were included in the analysis, as the number of respondents affected by Parkinson’s disease (237) was too small for meaningful analysis. Among the 1312 responses analysed, 102 had both epilepsy and Parkinson’s disease. The mean age of the respondents was 43 years. Respondents’ characteristics are provided in Table 1. Table 2 also shows manufacturer-reported shortages between January and April 2024.

Manufacturer-reported shortages were primarily observed for carbamazepine and lamotrigine during the study period, aligning with the medicines most frequently cited by survey respondents as being difficult to access.

### 3.2. Access to Medication

Difficulty in accessing medication

**Figure 1:**
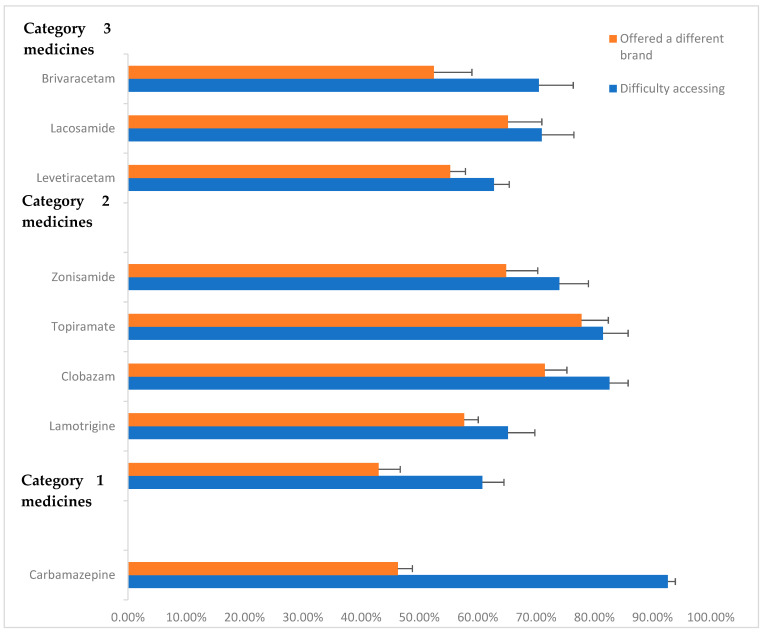
Proportion of respondents reporting problems with accessing ASM in previous year (2023) and being offered different brands. Standard error bars provided.

A total of 941 respondents (71.7%) reported having difficulties in accessing their antiseizure medication at least once over the past year. A total of 700 (53.4%) of the participants reported that they had been offered a different brand as a substitute for their usual antiseizure medication. We did not ask whether patients missed medications because of these difficulties. Figure 1 summarises the reported difficulties in accessing ASM in the year prior to the survey (2023) and the proportion of respondents who were offered a different brand as a result. Figure 1 presents the proportion of respondents who reported access difficulties and those who were offered a different brand, by ASM category.

The range of difficulties reported in accessing prescribed antiseizure medication as described in responses to the open-ended survey questions included receiving only part of the prescription, with pharmacies issuing “owing” notes and asking them to return for the remainder or being out-of-stock. In some cases, this led to brand substitutions, travel to alternative locations, or borrowing from other branches:

“*In the past I was given generic brand and told it was because of shortage. But after contacting manufacturer it turned out they had stopped supplying...*”.

For several participants, these challenges were not isolated incidents but represented ongoing or recurrent problems. Others described how such difficulties have persisted for years or worsened over time.

“*I have Monthly issue with eslicarbamazepine*”.

In a few cases, participants received medications of different strengths to what was prescribed, requiring them to adjust their dosage accordingly.

“*Different strength, 100 mg not 200 mg so have to take double amount*”.

In response to these ongoing challenges, several participants had adopted proactive strategies, such as requesting their medications earlier than usual:

“*I order mine straight after collection of my previous prescription and have a month’s supply ready.*”

Pharmacist support

A total of 750 (67.6%) of the participants reported that their pharmacist was supportive in accessing their medication during shortages.

The responses also revealed different levels of support offered by pharmacists during periods of medication shortages. Many participants expressed that their pharmacist was actively engaged in mitigating the impact of shortages. This included sourcing medications from alternative suppliers or branches, offering different formulations or strengths, advising on early reordering, and liaising with GPs to amend prescriptions.

“… *As time went on and the situation got more precarious, they did eventually contact my GP to ask them to prescribe the immediate release version as a backup*”.

Others noted being provided with partial supplies and owing notes to avoid treatment disruption.

Conversely, numerous participants reported minimal assistance. Pharmacists instructed respondents to try other pharmacies without offering specific guidance or support or simply told them to return later. A common statement respondents received from their pharmacist was as follows:

“*…I was given generic brand (Levetiracetam) and told it was because of shortage...*”.

In some cases, respondents were forced to contact multiple pharmacies independently or return to their GP for a new prescription. Due to these concerns some participants reported changing their pharmacy to be able to obtain the right medication on time.

Brand substitutions were a frequent source of concern, especially for those with seizure triggers linked to specific formulations. Some pharmacists disregarded requests for consistent brands, while others substituted strengths, requiring respondents to adjust dosing. This led to confusion, anxiety, and in some cases, seizure recurrence. Others reported delays due to their primary care prescriber not adding the brand of their ASM during prescription, leading to inappropriate brands being given and delays in obtaining the right ASM.

“*Difficulty obtaining consistency of brand and this can have an impact upon seizures*”.

Participants also highlighted poor communication as a recurring issue. Delays in pharmacists letting them know of shortages and a lack of follow-up meant some individuals ran out of medication. Others described inconsistent messaging and difficulties coordinating between GPs and pharmacies. Some also expressed that their pharmacist blamed “the government and wholesaler” for the shortage.

Several participants expressed distress and frustration at the perceived lack of urgency from pharmacy staff. For those with poorly controlled epilepsy, the uncertainty around medication availability was especially distressing:

“*They argue that from their perspective stocking my medication is too expensive and they will keep checking to see if they can get hold of it for me but from my perspective it feels like I am not a priority when it appears to be out of stock*…”

### 3.3. Impact of Antiseizure Medication Shortages

Number of times visiting multiple pharmacies and miles travelled.

Over the study period, 568 (43.3%) reported that they had never needed to visit multiple pharmacies to obtain their correct medication. However, 240 (18.3%) reported doing so on one occasion, 270 (20.6%) on two to three occasions, and 234 (17.8%) on more than three occasions in the last year. Table 3 shows the number of times respondents visited pharmacies and time travelled.

Content analysis revealed that many participants described being left to search for their ASM elsewhere alone when there are shortages, with pharmacists offering minimal support beyond returning the prescription during shortages.

“*…The favourite suggestion is get a paper prescription from GP and do a tour of pharmacies*”.

“*I have had to try 12 pharmacies before finding one that was able to get me the manufacturer of clobazam I needed*…”.

In some cases, participants described extreme measures to locate medication, including phoning pharmacies across the country or contacting suppliers directly:

“*Tried to get medication from all over the country and even Europe*”.

For those unable to drive, which is common for people with epilepsy, the burden was on friends and families to support them in collecting their medication.

“*…Also, as I can’t drive due to my epilepsy and the pharmacy was several miles away, and difficult to get to by public transport, I had to rely on a friend’s help to collect the medication*”.

The burden of visiting multiple pharmacies was particularly stressful for newly diagnosed patients and families.

“*…As newly diagnosed the stress of this has been magnified. Quite frankly the supply chain is a disgrace*.”

Respondents experiencing stress and/or anxiety as a result of a shortage of medication.

A total of 1028 participants (78.3%) reported experiencing stress and/or anxiety due to medication shortages.

Among participants taking sodium valproate, 116 (62.4%) reported such distress. For those on lamotrigine, 284 (66.0%,) experienced stress and/or anxiety, and 231 (64.2%) of those on levetiracetam reported the same. Notably, 350 participants (86.2%) prescribed carbamazepine reported stress and/or anxiety related to medication shortages. This was significantly greater than for other ASM (*p* < 0.001, Chi-squared).

Stress and/or anxiety caused by medication shortage resulting in seizure.

In total, 529 (40.4%) participants reported that stress and/or anxiety caused by medication shortages was associated with increased seizure frequency or severity.

## 4. Discussion

This is the first peer-reviewed study to describe the challenges faced by people with epilepsy and their caregivers in accessing antiseizure medications in the UK. The majority of participants reported experiencing difficulty obtaining their medication in the first quarter of 2024, frequently resulting in the need to change brands, travel significant distances, or visit multiple pharmacies. The impact of patients’ experiences on seizure control and quality of life remain unclear, although patients and caregivers associated medication-associated stress with worsened seizure control and severity.

Whilst challenges accessing all types of antiseizure medicines were reported, only two medicines were recorded as having supply difficulties on the national register by the pharmaceutical industry. This suggests that problems faced by patients are frequently occurring at the wholesaler and pharmacy level.

While these findings highlight a substantial burden, it is important to acknowledge a potential inherence bias; individuals who are more likely to experience short-term problems may be more willing to complete the survey, which could over-represent the prevalence of these challenges. Similarly, the inclusion of children in the response may provide inaccurate data as children do not collect their own prescription themselves.

Middle-aged adults (35–64 years) were the most represented respondents, contrasting with national epidemiological data showing a U-shaped prevalence pattern with higher rates in children and older adults [24]. The under-representation of the oldest age groups in this study may reflect recruitment biases, such as reliance on online/social media platforms, which are accessed less by older individuals. Future research could address this by adopting mixed-mode survey approaches, such as combining electronic and postal methods, to ensure broader participation and improve representativeness across age groups [21].

Whilst over 50% of people with epilepsy are reported to be men [24], there were more female respondents within the survey. This may reflect the fact that females are more likely to respond to surveys [25]. Again, the proportions closely resemble those within the members and supporters of the charity who achieved the greatest response (Epilepsy Action, personal communication, 18 July 2025).

The pattern of ASM prescribed in this study broadly aligns with UK prescribing trends reported in the NHS Prescription Cost Analysis (PCA) data [26]. Lamotrigine (29%), carbamazepine (16%), sodium valproate (17%), and levetiracetam (23%) (Table A1 in Appendix A shows the full data) were the most commonly reported ASMs, reflecting their dominant role in UK clinical practice. While our data shows clobazam is among the most frequently prescribed ASM for the participants, the PCA data reported a low percentage (3%), which may reflect differences in the study population, such as age distribution, seizure type, or regional prescribing practices of participants as this ASM is more commonly used in children [27]. These findings demonstrate that our sample is broadly representative of ASM prescribed in the UK and provide context for interpreting the experiences and challenges reported by people with epilepsy regarding medication access.

The UK Office for National Statistics (ONS) [28] reports that only 20% of patients in England face challenges accessing their medication; our study identified a much higher proportion among people with epilepsy. This discrepancy likely reflects the unique challenges associated with epilepsy, particularly the clinical requirement to remain on the same ASM and the limited flexibility in switching brands or formulations. In addition, a lack of awareness among non-specialist healthcare professionals, such as general practitioners and pharmacists, may contribute to this problem. Another explanation may relate to the recruitment pathway; people engaged with epilepsy charities are often those with more complex or refractory epilepsy who may require less commonly prescribed ASMs, whereas individuals whose seizures are well controlled are less likely to seek support from these organisations [29].

According to manufacturer reports, various forms of carbamazepine were out of stock between January and April 2024, which aligns with some of the experiences of participants prescribed this medication in our study [30]. Community pharmacies often rely on a small number of wholesalers, which enables them to maximise the discount they receive on purchased medicines by promising to purchase the bulk of their medicines through one wholesaler [31]. The other reason for limiting the number of wholesalers accessed is because the wholesaler and the pharmacy are sometimes in effect the same company, and this practice is allowed in the UK [32]. Finally, if a doctor chooses to prescribe a generic version of the medication, for which the pharmacist receives reimbursement at a set level, it means that the ‘usual’ medicine may not be supplied [33]. The high proportion of patients reporting problems accessing ASM not related to manufacturing problems suggests that at least some of the problem is likely to be either at the wholesaler or community pharmacy level.

The high rate of brand switching experienced within this study is concerning, as it may result in the risk of confusion, dosing errors, and potential breakthrough seizures [34], particularly given the narrow therapeutic index of many ASMs. Such substitutions are likely driven by factors including medication shortages, pharmacy substitution policies, and cost considerations [35,36]. Further research is required to identify and develop effective approaches to preventing the substitution of ASM brands by clinicians. In the UK context, initiatives such as Epilepsy Action’s Don’t Sub My Drug leaflet [37] provide an important resource to help patients understand what substitution is acceptable and how to verify that they are receiving the correct medication.

Community pharmacies, as private entities contracted to provide services to the NHS, determine their own wholesaler arrangements. In some cases, pharmacy companies are vertically integrated, with the pharmacy and wholesaler under the same ownership. This can restrict flexibility, as pharmacies may choose to purchase only from their own wholesaler, leaving limited options if a product goes out of stock. Data indicates that about 60% of community pharmacies rely on a single wholesaler [31], which highlights the potential vulnerability of the supply chain of ASMs in the UK. It is important to note that these supply chain situations are UK-specific.

Whilst wholesalers themselves can inadvertently go out of stock of certain products due to their approach to stock management, moves by pharmaceutical companies to provide quotas for their products to be supplied to wholesalers, to prevent resale abroad, have exacerbated the problem [32]. This largely occurs with high-cost branded medicines rather than generics, which may partially explain some of the problems seen, given that the prescription of antiseizure medicines is more likely to be via brand to ensure consistency.

The ONS data released in September 2024 revealed that 79.9% of people were satisfied with pharmacy services in England [28]. In contrast, the level of satisfaction with pharmacy services in this study is lower than that reported nationally, which may again be explained by the greater proportion of problems experienced in this patient group and the seeming inability of a number of pharmacies, in some situations, to address them. In many cases, pharmacists who were deemed unsupportive simply informed patients that their medication was “out of stock”, without further clarification or efforts to assist. These instances place the burden on individuals, which is especially problematic for those newly diagnosed or with limited access to transport. The General Pharmaceutical Council (GPhC), which regulates pharmacists in the UK, advises that when a pharmacy is unable to supply a particular medicine, they should communicate with the patient to discuss available options [38]. The pharmacists should consider contacting manufacturer(s) or checking their website for supply updates, using a Serious Shortage Protocol (SSP), where applicable to substitute the prescribed medicine, offering to contact the prescriber to jointly consider an alternative brand or medicine and checking the availability of the medicine at another local pharmacy [38].

The study also highlights the burden of shortages. A majority of the respondents reported needing to visit more than one pharmacy to receive their ASM. Also, about half travelled over 5 miles to receive their ASM. For those unable to drive, this caused additional stress on family and carers, therefore creating inequity in access to ASM. Participants frequently expressed frustration at the lack of urgency perceived among pharmacy staff, and many described taking matters into their own hands such as stockpiling or reordering early to protect against future disruptions. However, such behaviour potentially exacerbates the problem [39], as observed during the COVID-19 pandemic, where increased demand and panic buying contributed to worsened medicine shortages [40]. In resolving this, pharmacists and suppliers should implement real-time stock-tracking systems to alert patients to ASM availability. Also, provisions should be made for home delivery services for patients with limited mobility.

Most concerning is the psychological impact, with two thirds of participants experiencing stress or anxiety due to medication shortages and one third believing that the shortage of their ASM contributed to seizure reoccurrence. Other studies have expressed similar concerns, as patients not receiving a sufficient supply of ASMs is a predictor of non-adherence, leading to poor seizure outcomes [41]. Another study revealed that ASM shortages may impact clinical outcomes such as compromising treatment and causing delay in treatment [42]. Poor clinical outcomes for people with epilepsy results in worsening seizure control, which can increase the risk of Sudden Unexpected Death in Epilepsy (SUDEP) [43]. About 20% of people with epilepsy visit the emergency department annually [44]. Although national data quantifying emergency attendances directly attributable to medication alterations caused by shortages are unavailable, seizure recurrence resulting from such changes is likely to contribute to avoidable hospital presentations. In this study, some participants reported that their ASMs were switched due to unavailability. Previous research has shown that brand switching can double the risk of seizure recurrence [45]; however, the present study relied on patients to make the link between brand switching and seizure control.

### 4.1. Strengths and Limitations

This study was co-designed with patients and piloted to optimise face validity. This study used a range of methods to optimise response rates including advertising through charity newsletters, social media, and email communications, which helped to achieve a large response. However, as the total number who received the questionnaire is unknown, the response rate could not be calculated, making it unclear how representative the respondents are of the wider population and potentially affecting the generalisability of the findings. Due to non-random sampling, the response rate does not affect the depth of quantitative and qualitative results, but caution should be exercised toward extrapolating the results to all patients with epilepsy. The respondents represent a self-selected sample, primarily drawn from individuals engaged with an epilepsy charity, and may not represent the broader population. This group is likely to be more actively engaged in the care of patients with epilepsy and reasonably well educated given the methods used for distribution and completion. Again, as the study relied entirely on self-reported data, there is potential for recall and social desirability biases. There is the potential for response bias, including overexaggerating the problem to encourage government action. A major limitation was that we neither collected data on actual ASM alteration such as missed or reduced doses nor collected any data on seizure rates; hence, the study could not link medication changes with seizure recurrence. 

### 4.2. Recommendations

These findings reinforce the urgent need for strengthening available system-wide strategies to address ASM shortages in epilepsy care. Further research is required to identify and develop effective approaches to preventing the substitution of ASM brands by clinicians. Also, future research should collect detailed data on ASM administration to directly compare medication alteration and seizure reoccurrence. Furthermore, future research should consider combining data from advocacy groups with clinic- or registry-based recruitment to reduce response bias. To mitigate shortages, it is recommended that a dedicated emergency reserve of critical medicines, including ASM, be established similarly to the NHS emergency inventory system to ensure continuity of supply during shortages. Lastly, future research should employ primary care networks and utilise social media platforms to capture a broader spectrum of experiences.

## 5. Conclusions

This study identified that shortages of ASMs caused stress and/or anxiety, resulting in people with epilepsy experiencing recurrent seizures. For people living with epilepsy, medication continuity is not a matter of convenience but a critical element of seizure control, safety, and quality of life. The higher level of problems reported by people prescribed ASMs and lower levels of satisfaction with pharmacy support, compared to national figures, demonstrates the unique problems experienced by this patient group due to the need to remain on the same ASM. A significant proportion of problems exist at wholesaler and pharmacy level, and currently we can only postulate as to the most likely causes. Further research is required to identify the exact causes underpinning the problem and thereby enable legislative, regulatory, or organisational changes to be made. The high proportion of patients offered alternative ASMs where this may not be appropriate or ideal also warrants further exploration to enable the identification of approaches to prevent this.

## Figures and Tables

**Table 1:** Characteristics of respondents.

All	Category	Count (%)
Age	0–17	139 (11.3)
	18–24	93 (7.5)
	25–34	177 (14.4)
	35–44	213 (17.3)
	45–54	239 (19.4)
	55–64	209 (17.0)
	65–74	115 (9.3)
	75–84	42 (3.4)
	85+	6 (0.5)
	Missing	79 (6.0)
Gender	Male	389 (29.6)
	Female	836 (63.7)
	Other	8 (0.6)
	Missing	79 (6.0)
Questionnaire responder	Self	896 (72.9)
	Caregiver	333 (27.1)
	Missing	83 (6.3)
ASM prescribed	Lamotrigine	417 (31.8)
	Carbamazepine	400 (30.5)
	Levetiracetam	357 (27.2)
	Sodium Valproate	177 (13.5)
	Clobazam	140 (10.7)
	Topiramate	80 (6.1)
	Zonisamide	77 (5.9)
	Lacosamide	67 (5.1)
	Brivaracetam	59 (4.5)

**Table 2:** Manufacturer-reported shortages between January and April 2024 (source: Epilepsy Action. “Drugwatch” 2025, available at https://www.epilepsy.org.uk/news/category/drugwatch (accessed on 21 August 2025) [22].

Drug	Date of Reported Shortages
Carbamazepine (Tegretol Prolonged Release 200 mg and 400 mg tablets)	9 January 2024–15 February 2024
Carbamazepine (Tegretol Prolonged Release 400 mg tablets)	25 January 2024–15 February 2024
Lamotrigine	5 February 2024

**Table 3:** Number of times visiting multiple pharmacies and miles travelled.

Impact	Number No. (%)				
	Total	Option 1	Option 2	Option 3	Option 4
No. visiting multiple pharmacies	1312	Never	One occasion	Two to three occasions	More than three occasions
		568 (43.3%)	240 (18.3%)	270 (20.6%)	234 (17.8%)
No. pharmacies visited	741	One pharmacy	Two to five pharmacies	Six to nine pharmacies	Ten or more pharmacies
		98 (13.2%)	517(69.8%)	75 (10.1%)	51 (6.9%)
Miles travelled	1260	Up to 5 miles	5–10 miles	10–20 miles	More than 20 miles
		302 (24.0%)	217 (17.2%)	102 (8.1%)	64 (5.1%)

## Data Availability

The data presented in this study are available on request from the corresponding author due to ethical reasons. Manufacturer-reported shortage data are available at “https://www.epilepsy.org.uk/news/category/drugwatch” (Accessed on 9 September 2025). Prescribing data is available at “OpenPrescribing.net” (Accessed on 9 September 2025).

## Supplementary Materials

The following supporting information can be downloaded at: https://www.mdpi.com/article/10.3390/pharmacy13060166/s1.

## Appendix A

**Table A1:** Prescribing data between January and April (source: “OpenPrescribing.net” (Accessed on 21 August 2025).

Drug	January	February	March	April	Total	Percentage
Sodium Valproate	193,012	181,886	181,186	186,565	742,649	17%
Carbamazepine	174,128	175,197	175,332	179,403	704,060	16%
Lamotrigine	333,173	314,938	319,034	331,929	1,299,074	29%
Levetiracetam	261,358	247,057	251,442	260,880	1,020,737	23%
Clobazam	33,527	31,762	32,306	33,679	131,274	3%
Topiramate	100,515	95,413	97,320	99,948	393,196	9%
Zonisamide	16,467	16,233	15,901	16,605	65,206	1%
Lacosamide	22,848	21,823	22,187	23,529	90,387	2%
Brivaracetam	9728	9288	9635	10,246	38,897	1%
					4,485,480	
