# Supplementary material for: Experiences Reported by People with Epilepsy During Antiseizure Medication Shortages in the UK: A Cross-Sectional Survey
Source: Pharmacy (Basel). 2025 Nov 10;13(6):166. doi: 10.3390/pharmacy13060166 (PMC12641818; doi:10.3390/pharmacy13060166)
Supplement: Supplementary file 1 [file pharmacy-13-00166-s001.zip › content analysis of difficulty in access S1.pdf]

Content analysis of difficulty in access S1

Name: (no concern)

I still take medication in full even though seizure free.

Had the pharmacist at my doctors for many years 50yrs

Getting the correct brand

Name: change of brand

Receiving different brands

I keep having different brands of the same medication & as my condition isn't fully controlled, I don't know if this is having any impact

I haven’t been able to get the same brand I normally do

Each prescription differs every 2 months

Difficulty obtaining consistency of brand and this can have an impact upon seizures

Difference brands every time including one with no English on the package

Constant changes of brand can be very confusing. The lamotrigine sometimes has 100 mg tablets that are tiny and 50 mg one considerably bigger

Change of brand

Been getting them ok but not usual brands

All cheaper brands are being used as replacements

Content analysis of difficulty in access S1

Name: change of meds

I have been taken off the liquid medication that I was on because it was deemed too expensive

Name: changing of dose

Had to change dosage

Different strength. 100mg not 200mg so have to take double amount.

Name: check other pharmacy

Sometimes takes longer as the chemist aren't getting them in often I have a barcode to get them elsewhere

Ive managed to get my medication but have had to return to pharmacy multiple times when they tell me about a delay

I have had to go to another pharmacy on a couple of occasions

Borrowed from another store

Name: early request

My pharmacist has advised me to request my medication earlier than usual

I order mine straight after collection of my previous prescription and have a months supply ready

Name: insurance issues

Yes my insurance stop

Content analysis of difficulty in access S1

Name: issue persists

Unable to get rasagline.

Not able to get Keppra which is what my GP wants me to have

Monthly issue with Eslicarbamazepine

Latest prescription and about 4 months ago

it is taking longer to get the prescription- it used to be ready within 3 days but now it is usually 6-7 days

it is happening on a regular basis

It is an ongoing issue

in the last month and a few months ago

in fact over the last 3-4 yrs

I had trouble three weeks ago and a couple of times in the last few months

I couldn't get any tegretol slow release

Every time l put in for repeat prescription

Every time

Every refill there is a problem

During and after Covid

Difficulty for a couple of years and worse in the last 6 months

At the moment

Content analysis of difficulty in access S1

also had problems before period asked about. Problems grew after Brexit 1

Name: lack of knowledge

However, I do not know if generic medication has been used

Name: Medication out of stock

Very seldom, some meds are out of stock

Previous months prescription. But my husband travelled to a different county and got them

In the past I was given generic brand and told it was because of shortage. But after contacting manufacturer it turned out they had stopped supplying Tesco and Boots .

Name: miscommunication between healthcare provider

Miscommunication between neurologist & pharmacy for days

Name: owing medicine by pharmacy

the pharmacy that i get my medication from regularly give me an owing note for my Epilim

Just getting half and going back to the chemist

Name: request emergency supply

Last year it was difficult getting my Phenytoin sodium Flynn, when none of the pharmacies had any while I was down to my last 2 days, and then I struggled to even get an emergency supply, and then I had to ask for yet another dam prescription and after trying for a long time I hot them in the end. The pharmacy had to order some when I got the emergency supply

Content analysis of difficulty in access S1

Name: stress reordering meds

HATE HAFVING TO REORDER ITSBECOMMING STRESSFUL

Name: use independent pharmacy no problems

I have had no problems so far

Name: visit pharmacy severally

He had to go back twice to get all of prescription

Name: wrong brand prescription

Last year I was given a brand that was not keppra. I requested my gp switch the prescription back. They said it may not be in stock consistently and this was to make sure I get the same drug each time. I didn't want that because I know even swapping brands of the same drug can trigger seizures and worse side effects. I refused it and they switched it back to keppra!
