# Supplementary material for: Experiences Reported by People with Epilepsy During Antiseizure Medication Shortages in the UK: A Cross-Sectional Survey
Source: Pharmacy (Basel). 2025 Nov 10;13(6):166. doi: 10.3390/pharmacy13060166 (PMC12641818; doi:10.3390/pharmacy13060166)
Supplement: Supplementary file 1 [file pharmacy-13-00166-s001.zip › Content analysis of pharmacy support S2.pdf]

Content analysis of pharmacy support S2

Name: added alternative meds

Very difficult, had to have 2 separate prescription alternatives added to medication

"The initial response of my usual pharmacy was just to say we can't get it and we don't know when we will. As time went on and the situation got more precarious they did eventually contact my GP to ask them to prescribe the immediate release version as a back up.

Gave advice on alternative options

Doctors surgery modified prescription prior to pharmacy

Name: blame the government

"They just blame the government.

Name: blame wholesaler

They blame their wholesaler

Name: Cannot reorder

Just been told they cannot re order from Novartis. Until today they had some in but they still cannot re order it in! I was so surprised I didn't ask where the boxes found had come from!

Name: change medication

Yes when after many times of saying I can't go to dr and just change my medication.

"On the whole no, I had to make a lots of phone calls to try and find medication myself. A pharmacist eventually contacted the manufacturers to find out when it would be available. We had to delay starting a new medication and weaning plan due to not being able to acess the correct medication.

Content analysis of pharmacy support S2

My local pharmacist telephoned other local pharmacies to be able to fulfil my prescription and was able to give me a slightly different medication to tide me over

My daughter's pharmacist gave her completely the wrong medication. This resulted in her having severe seizures after being seizure-free for 6 months. Her pharmacist does not make an effort to get her the correct drugs, leaving me to search around every 8 weeks for the correct medication

Local pharmacy now refuses to supply medication as it expensive

Name: change of brand

Unfortunately 3 different brands, unacceptable

Tried to substitute with generic brand rather than named brand

Tried hard and all others locally. Had to swap to another make and then the next time too. I'm not meant to keep changing manufacturers

They tried so hard to secure it. He, the pharmacist made us aware there seems to be an issue coming up. We thought we might have enough to see us through. But we didn’t and had to transfer into another topiramate

They tell me to pick another one as if i was selecting a chocolate bar rather than collecting my medication

They struggled to get hold of the Accord brand and tried telling me brand didn't matter, even though I told them otherwise. I believe the system then changed and they told me they couldn't specify brand when ordering medication anymore

"They looked for other suppliers and types of this medicine eg liquid form, so it was more accessible

Content analysis of pharmacy support S2

"They haven't been calling me to update if there are any in or if they're trying to help me find medication. They're rude and advised me to change my medication but they don't understand the anxiety this also has with changing when my body is so used to the ones I am on. I am having to skip my medication to ensure I don't run out which is having impacts on side effects to a seizure or absences

"They don't engage with me at all, they change my brand all the time, which causes problems. But as I'm banned from all other NHS wales care and visiting my GP in Wales, there's nothing I can do about it

They are giving out the cheaper brand which not as effective

There was shortage of Topamax at my local pharmacy so pharmacist tried to source some from other their other branches. He was unable to find any so offered me an unbranded version instead. I was reluctant to take this as I had been given it previously and suffered breakthrough seizures/nausea. So he gave me the prescription so I could try to get it from another pharmacy. After contacting several local pharmacies with no luck I contacted my Epilepsy Nurse to see if she could help. But there no stock at the hospital either. At that point I was starting to panic, so I tried calling another local pharmacy, who said they had some Topamax in stock. But when I went to collect I was given a branded version instead. The pharmacist obviously didn't fully understand the difference between the branded and unbranded versions and the dangers of interchanging them. I felt had no option but to take these rather than have no meds at all. The whole experience was extremely stressful and I was constantly anxious it could trigger a seizure

The pharmacist has and as I've seen multiple in my chemist they have said they don't want me changing brands or names on boxes or styles, colours, shapes etc. The people in the chemist who work there are a problem where they don't seem to care which I have complained about.

"the one pharmacist is lovely and will try to ring around to their other branches. if I cannot get them at their other branch which is 2 miles away then I have to wait until they are in stock and the ones I am given are often looking very different and of generic looking made.

"The majority of pharmacists were not supportive. My first experience the pharmacist strongly advised I switch to the generic brand of lamotrigine due to the shortages of

Lamictal. At the time I was pregnant, and so the risk of seizures was far higher and the repercussions far more dangerous. I had to have a lot of conversations with different pharmacists until I spoke to one pharmacist who was willing to advocate for me and ensure I only received Lamictal from then on. He was incredibly helpful and ensured my GP changed my prescription to request only Lamictal. He was the only pharmacist who was willing to help.   My second experience was with a shortage of a particular dosage (I require 100mg and 25mg of Lamictal). There was a shortage of 25mg so I was told I might have to order the 50mg and split them in half at home. I found this ridiculous given the shape of the tablets there is absolutely no way I could do this. Putting this responsibility on me instead of the pharmacy could potentially have been damaging. As a result I have been ordering my prescriptions weeks in advance so that the pharmacy can order it in time. I can never collect my prescription straight away, I have to wait weeks while they specifically order each medication. I am regularly anxious that shortages could affect my epilepsy. Unfortunately the majority of pharmacists still insist that there is no difference between the brands, even where I have seen evidence provided by epilepsy charities and my epilepsy nurse that this is incorrect.

"The dispensary at my surgery have not been very helpful but the Boots branch has although I have had to change brand which I know is not advisable

"The dispensary at my surgery have been very supportive , giving a prescription sometimes : but not understanding when I asked for a particular brand. The nearby branch of Boots have been helpful and I have spoken to the pharmacist there. Both pharmacies have had to order in and I have had to change brands which I know is not advisable

"The brands keep changing but there is always supply

"Swapped trihexyphenidyl for tablets from liquid, but she’s on ketogenic diet for her epilepsy and this messes with her carb intake (she is fully tube fed), plus crushing tablets is a blockage risk.

"Suggested I look for lamictal instead

"Sort of! Our alternative is to get some direct from the hospital 40 mins away or dissolve more lamotrigine than is required to get the right dose ie dissolve 50 to then draw up dose of 35

"Pharmacy tries to get the right brand but sometimes sends PI even though my prescription is clearly UK only no PI

"Pharmacy offered different much cheaper generic brand disregarding the fact that it caused severe side effects to me. I don't drive and had to travel to another town to seek my medication. GP would issue repeat prescription only a week prior running out of meds. This circus repeats every 28 days. The stress exacerbates risks of having seizures.

"Pharmacy 2 u just started sending a different brand out of the blue. Called Chalfont Centre and was told by my neurologist NOT to switch brands. I bounce between local chemist and them.

"Pharmacists are ignoring brand requests which can have a detrimental impact on the patient.

"Pharmacist has offered to give me original prescription when pharmacy has provided substituted drugs but could not guarantee source of other pharmacy supliers.

"Pharmacist has had to ring round the area to access my prescription on one occasion after not being able to get a delivery. On another there was none available locally but they could get 400mg tablets (I take 200mg) so they kindly halved these for me so that I could continue with my prescription.

"Ordering correct brand multiple times but alternative sent each time

"Online pharmacy initially sent generic brand, but engaged in email and phone conversations concerning this, and on one occasion released the reference so I could obtain the one remaining box in local pharmacy.

"Offering different brand

Content analysis of pharmacy support S2

"Offered another make but can’t take it as it’s not compatible

"Offered alternative ""makes"", e.g. generic

"Not very helpful at first a couple of years ago at getting correct Lamictal but became more helpful

Not really but amazing when I challenged them about when & if it was amazing that the Tegretol Prolonged Release 200mg tablets (Novartis Pharmaceuticals UK Ltd) turned up within the next two days but they never contacted me it was only because I picked up another prescription that the Tegretol was in the bag. The chemist never rang to tell me it was in. Total disgrace.

"No so much as not getting any but mixing the brands

"No pharmacy 2 u often cannot get it so I go to the local pharmacy who usually have it although we’ve had to change brand

My pharmacy is very helpful, with it being connected to my GP practice, it does make communication easier. However they decided to put me on a tad more expensive version if my carbamazepine. I was on Tegretol Carbamazepine up until January but had to be moved onto Medreich Carbamazepine

My pharmacist managed to persuade my doctor to prescribe a different brand

Wrong brand of medication which required returning back to pharmacists via courier and my actual medication being reissued

Mixing medication brands, loads of notices - and because of the constant brand changes, always being asked if I’m on contraception, cross checking with other meds and making it really difficult to just get the medication with jt approval from senior pharmasksts

Lamotrogine has had to be sourced from different providers so we can't have the brand Accord which is on our prescription. Clobazam has been difficult to source earlier in the year, again different brand.

Lamotrigine Accord 25mg tablets are currently long-term unavailable. My wife has to take Lamictal 25mg tablets instead which we suspect affect her mobility more than Lamotrigine Accord.

I've just explained and insisted on sticking to one supply of the same meds or go else where.

It was not a shortage, it was trying to obtain a particular brand Ethosuximide.

Initial pharmacy didn't provide any update, since swapped to a different one & much happier with the service.

I was given a different brand which I didn't like

I usually take generics of all three anticonvulsants but my pharmacist inputs an official exemption and refills my prescription with brand-name medications instead when there is a shortage.

I tend to assume that they are getting whatever brand they can

I pick up my prescription weekly and lately I've only been getting my antiperspirants only in my bag. No Eplim Chrono which I need daily.. I have to ring doctors and receptionist will say I don't understand why it hasn't gone through. Last week my chemist closes at 5.30pm, and by the time I'd been on phone to doctors my pharmacy was closed and prescription had to be sent to another pharmacy. I got there, receptionist said she'd send it to the chemist. I got there and nothing was there.

I have used and still use my local pharmacist for over 30 years to access phenobarbital (accord). Now I am having problems accessing it. Told by annoyed pharmacist and assistant regularly that they could only be given different brand. Have seizures when switch brands. Told by pharmacist different brand worked the same, pharmacist trying to give me different brand, which I refused. It does not work the same. Eventually, after several stressful months re accessing accord phenobarbital 30mg, told by different pharmacist name of

brand needed to be on prescription. Phoned Accord who said there was no shortage of phenobarbital 30 mg. G.p. changed prescription to have Accord written on phenobarbital prescription. Pharmacist, who told me Accord needed to be on prescription, at that time refused prescription. Have needed to go to other pharmacist an hours bus ride away to access it. Finding this very stressful, stress being one of seizure triggers. I still regularly get email /message from pharmacist I use for over thirty years saying need to discuss the phenobarbital prescription. I am now ignoring them for a while and the mediction is coming through at moment. Not sure what they are wanting to discuss as I have said to pharmacist I cannot change this and g.p. has this on my notes.

I have been told not many people are taking this product so just accept the mixed batches from the companies they can supply my prescription from which is actually not supplying my prescription which clearly states brand medication due to coloring allergies.

have been prescribed alternative medicine until the issue can be resolved

I have a sensitivity to certain brands (Possibly their sugar coating), which leads to increased seizures. Pharmacist is aware and tries to source one I'm not sensitive to

I had to switch to Keppra because my usual brand (Accord) was continually out of stock). They phoned round other Kamson's and ended up sending some from further north down for me. Even then it was only half my supply.

I had to go to the doctors to tell them because they could only get the expensive one topamax and need there approval, so now when I take the prescription to the doctors they have to find out which one the chemist can get to order it

I am prescribed the â€˜Accord' brand of lamotrigine but this has not been available. My pharmacist has been able to access other brands (but this change has caused me some anxiety).

However it was suggested that I could have a different Brand of the medication. I declined after speaking to someone at Epilepsy Action and being told that even changing brand could potentially cause problems.

Content analysis of pharmacy support S2

He was ringing the suppliers every day although he did just offer me just tegretol which has resulted in me having seizures in the past

Have to always write on the bottom of prescriptions don'tgive my daughter the wrong brand as it gives her belly aches

Had to go other pharmacies and get different medication that were similar to my prescribed tablets as mine were unavailable!

First pharmacy tried swapping brands and hoped we wouldn,t say anything. Wouldn't accept that brands couldn,t be swapped.   Changed pharmacy and they have tried their best but struggled many times had to travel round looking for the medication and sometimes was close to running out

First pharmacist tried to give me different brand of phenobarbital, saying drug was same in different make. Switching brands causes seizures. Months of stress and unfriendly pharmacist and shop assistant. Finally another pharmacist advised needed to have brand name written on prescription by g.p. to access brand I wanted. Had not had problems for thirty years accessing phenobarbital brand I needed. Brand Accord. G.p. wrote Accord on future prescriptions for phenobarbital. First prescription refused by pharmacist who had advised me to get Accord put on prescription. Had spoken with Accord who said there was no shortage of phenobarbital in area. Had to travel by bus to other pharmacist a distance away to access medication. Each month continues to be stressful re accessing phenobarbital.

Explained I would have a change in supplier and name

Can't get brand have had to use generic, then had to wait for over a week for it to be brought in from elsewhere

But unable to get the correct brand

Branded medication unavailable (Rosemont Phenobarbital) pharmastist asked me to write a letter for the pharmaceutical company to say I understood the implications of not having the Rosemont brand and I would accept whatever they offered   It was a ridiculous situation if I didn'tget the medication my daughter would have been in ICU

Because they are named brands, they have been hard to get together.   I have to name/nominate a chemists to which the prescriptions will be sent. Finding one that can get

either or both has proven difficult. If the named brand is dropped for a generic version, they can sometimes get one but not both. Lloyds, who were my nominated chemist, were brilliant. Never letting me leave with anything other than what the prescription said. However they closed, and finding another that could get both was nigh on impossible. 18 different ones tried before finding one that could. Then after the first month, I'd to start searching again. Going as far as 15 miles trying to find one. Then found that ASDA could get them, and apart from two occasions which they've corrected I've been lucky since then. Having been put in hospital due to generic medications being supplied, I'm not keen on taking something that will put in there.

Name: change of dose

Yesterday when refilling our son's pill container I found out we'd run out Oxcarbazepine. He had plenty of the rest of his medication so I can only assume we hadn't been given enough last month. I went to our local pharmacy to find they were shut for Easter. I did an emergency prescription on line and had it sent to the pharmacy at our local Tesco. I went down to Tesco with a letter from our Son's epilepsy consultant with his current dose, explained my predicament to the Pharmacist and after a little wait was given an emergency weeks supply of Oxcarbazepine.   Our son takes 750mg of Oxcarbazepine twice a day. He takes this as two 300mg tablets and 1 x 150mg tablet. Our local pharmacist couldn't get hold of 150mg tablets so they gave us extra 300mg tablets and told us we could break them in half.

When I order the medication they struggle with getting the right dose and suggest to change to a different two tablets that it will make it to the equivalent amount as the supplier is struggling to make the one tablet .

When 500mg tablets where unavailabile my pharmacist got my medication Changed to 300mg and 200 mg tablets

We have had shortages in Zonegran 100mg so they have only been able to give us 50mg so we have had to make the doses up in smaller doses

Unable to get 100mg tegretol so he split 200mg tablets for me.

"They struggled to get 200mg tablets and suggested Idoubled up on 100mg tablets instead

"They have phoned through to other pharmacies when I have needed some or they have given me other strength tablets to take

"They halved the 400mg tablets to give me my 200mg dose twice a day

"They gave me a half dose with instruction to take 4 a day rather than

"They didn't to start with but as soib as I made then Aeare they sourced double the strength and gekd then back for us.

"They can't get my dose so are providing me with 100mg tablets, I just have more to swallow

"They are trying to change doses but go very slow on changing prescription

"So-so, with my mom calling and pushing Ins Co go approve different dosage to make up for shortage

"SHE IS HAVING TO GIVE ME 100mg TEGRETOL BECAUSE OF SHORTAGES BUT DRS ONLY SDUPPLYING ENOUGH FOR A MONTH AND THEY ARE POSTDATING MY DIAZEPAM ALL THE TIME.

"Pharmacy have sourced smaller doses of medication but this affects compliance having to take increased number of tablets

"Pharmacy couldn’t get hold of 500mg and tried to get me to move to another brand. I’ve tried other brands before but I have side effects. I asked if I could have 250mg and take 2 tablets. And the reply I got was but then we’d have to give you more tablets. I don’tsee what the issue is.

Content analysis of pharmacy support S2

"Phamacist is 60mile round trip. Went to collect 3 boxes Clobazam (CLO) at Xmas, got given 1 card. No Tegretol(CBZ) 400 at all last month. Have cut CLO from 30mg/d to 20 and CBZ from 1200mg/d to 900 7.

Perampanel was only available in 2mg tablets so I had to get a new prescription from the doctor taking double 2mg. tablets instead of 1 4mg. tablet which had been prescribed

"one time they didn'thave my correct dose im on 750mg twice a day and they gave me 500mg instead as they said they couldn't get my 750mg

My pharmacist rang me to inform me of the shortage and said they had leased with my doctor, and found some 200mg tablets to have in place of the 400mg. They have said they will ring me to keep me updated too.

. Wrong dosages being issued

Instead of giving me Carbemazepine 400mg tablets.The pharmacist managed to get 200mg

I've had a shortage in Carbamazepine I normally take 200mg morning and evening they couldn't get my prescription of 200mg pills so unfortunately they only way around it was contacting my doctor and they said to get the 400mg and cut it in half

I take 400mg of Tegretol, then they gave me 200mg and just told me to double and then they run out of all and don't really tell me anything and I just had to wait so a bit of yes and no

I normally take 200mg morning and night but had to get 400mg and cut them in half

I have had two pharmacists ask if I 'still had epilepsy'. I have never had any attempt to make sure the medication is in stock in spite of its being a regular order from the same pharmacy. Topiramate is the problem medication and I take it at two doses - I have been told to double the lower dose, use a pill cutter, etc., which is not an issue in the short term but then there is no communication from the pharmacy to my GP and when I inevitably run short early, my GP queries the refill.

I have had trouble getting Tegretol 400mg and have often been given a load of 200mgs instead. I now allow at least a week for the prescription to be ready and have, on occasion, only been given part of my prescription and asked to return in a few days time for the rest.

He has had great difficulty in finding any Topiramate 50mg. Therefore he has had to replace them with 25mg Topiramate. These tablets are small and white and I am grateful to get them, however I am on other medications which are also small and white and it is sometimes confusing when taking my tablets.

Has provided other quantities of medication

Had to go to GP to alter prescription to correct amount of 100mg as 200mg not been available in my usual brand. Kept being offered alternatives

Cant blame pharmacy but couldn't get Tegretol Prolonged Releases 400mg on repeat prescription 18.03.2024 submitted, and pharmacy still don't have any. This is my main drug for my condition and struggled to get 200mg 2 months ago but eventually got.

But slow at telling me if there is no supply   And then slow to get supply   Changed the mg

Boots would not give me 100mg instead of 200mg

Although I couldn't get Tegretol in my home town. The pharmacist offered a different strength for me that I'd adjust the amount of tablets I needed. I decided against that, and I found some Tegretol in the next town, 10 miles away

Name: change of formulation

"They gave me liquid carbamazepine instead

Let's you know when meds in. Advice to try capsule instead of tablet for Topomax

I opted to have ordinary Tegretol instead of tegretol retard until supplies arrived. Living in a village the doctors dispenses medication so it could easily be approved by a gp

He just gave a syrup

First pharmacy was not helpful. Had to change to liquid from tablets

Content analysis of pharmacy support S2

Difficulty obtained Tegretol Prolonged Release and having to swap to liquid formulation

At first left me with just .out of stock.then after I said can't be without them.I have been given liquid to take 4x daily

Name: change pharmacy

When shortage Tesco they where not at all helpful which led me to phone Walton Centre who gave me a letter stating how important it was to get the same brand

To start with no, so moved to helpful pharmacy

They just tell you to go to another pharmacy

"The Pharmacist could not source it as they had only one supplier so I went to another pharmacy who was able to source it because they have several suppliers.   I have since moved to said pharmacy

"The pharmacist complained that it costs them Â£3 every time they get this medication for my son and that the order had been cancelled by the wholesalers. (I have since changed pharmacists)

"Our normal pharmacy was nof helpful. We have had to change pharmacy to get the medication. My mum now has to drive somewhere to collect it for me.

"One pharmacist was so rude I switched pharmacies to one further away. The new one is supportive.

My usual pharmacy has had wholesaler/supplier shortages specifically relating to 2mg ropinirole. I have had to use another pharmacy for those particular tablets.

Content analysis of pharmacy support S2

My daughters chemist just kept giving me her prescription back saying can't get these try another pharmacy 4 pharmacy s later we found a real helpful one. But still a struggle he said to get them.

My current local pharmacist I have only recently changed to has been fantastic. I can't say the same for Asda where I used to go, they were less than helpful.

I have had to change pharmacy to help

I have had to change pharmacy and although the smaller independent pharmarcys seem more likely to try and assist following up getting them I've had to switch to a larger pharmacy because Im currently on a newer medication and difficulties with it being in stock cos of of costs to them I think. They are also not ever available on the phones at Boots pharmarcy when they say they will be so you just cant get hold of anyone.

I changed pharmacy in the end as they didn't let gp surgery or myself no couldn't get it in the first place and never kept updated on I they could find it

Have now changed pharmacy.

Had to go elsewhere who were more helpful

Changed pharmacist from boots to Tesco, Boots were not very helpful with my medication kept expecting me to take brands which caused massive side effects.

Name: check other pharmacies

They just tell you to go to another pharmacy

"They just say try somewhere

"They have tried to find sources of Tegretol CR have managed to find it elsewhere by hunting around but recently had further issues

They have just said that they don’t have it and not provided an alternative other than a different brand, which I can’t have. However when this happened in the last couple of weeks they have now sent a code for me to access my prescription at a pharmacy in person.

"They gave me the prescription to try elsewhere and said if no joy to come back and they will grt doctor to prescribe a different medicine

"They found a small quantity at one of their suppliers.

"They are unable to give me any indication as to when stock is available. My gp is difficult to get hold of so I can access a pharmacy in Hertfordshire that does have stock.

"There were issues giving me the prescription to try at another pharmacy - it was ridiculous - in the end the hospital found me enough to see me through

"There is not much they can do. The favourite suggestion is get a paper prescription from GP and do a tour of pharmacies.

"The pharmacy releases the prescription back to the spine and we then need to ring round to see which pharmacy has it in stock. The shortages (which also affect my husband’s heart medications) must add to pharmacy workloads and previously helpful staff are often much less so. It is a time waster for us too, as our search also sometimes involves the GP prescription team.

"The pharmacy could not help but our drs in-house pharmacist called around and found a chemist that had medication in stock

"The pharmacist simply told me there is a supply issue with my medication and they couldn’t get it. Suggested I contacted other pharmacies to see if they had any stock.

"The pharmacist rang around several other chemists to get my phenytoin for me.

Content analysis of pharmacy support S2

"The pharmacist has been trying other pharmacies to get primidone and also spoken to my mums GP. However my mum has often been on her last few tablets and I have been stressed worrying that they wouldn’t be able to get them. My mum also has memory issues and so I have had to be in regular contact with her pharmacy to ensure her primidone is there when she collects her prescription

"The independent pharmacist repeatedly said there was none available and there was nothing they could do before I ordered my last prescription and then when I tried to collect. I waited a week then tried again, whereupon they said that the GP had not in fact sent the prescription, at which point I got a paper copy directly from my GP and went to Boots in Bradford, where they were able to supply a month's worth

"The advised which pharmacy could help

"Tesco pharmacy have said sorry, so we ended in calling inpatients pharmacy Cambridge and Peterborough luckily they helped especially big thanks to Cambridge who shared they medicine just before Christmas

"Sometimes they will try to get it from another branch. This isn't always possible.

"Sometimes it depends on the dose , particularly for small dose tablets such 150mg or 250mg...Sadly the pharmacist is resigned to there is a shortage and therefore says its difficult, will take longer and if not available suggests you go elsewhere

"Sometimes but you have to just ring every pharmacy until you manage to find a few and are on a constant hunt

"So I changed pharmacy

"Small local pharmacy has not had my medication in stock since Januaury. Closest Boots did not have it either. However, they sourced it for me at a different branch of Boots which I had to drive to. Luckily I am able to drive with my epilepsy so I was able to collect it easily.

Content analysis of pharmacy support S2

"Rang several branches to see if they had the medication in stock.

Pharmacy said I need go other chemist and try get it myself 15 Pharmacy and still could not get it very stressful and I get stress related seizures so this did not help has I have come really sensitive to epileptic medication which has caused me have seizures and a cardiac arrest so I need a specific brand which struggling to get

"Pharmacist suggested I went to another pharmacy

"Pharmacist called around neighbouring pharmacies to try and find medication

"Our pharmacist phoned round local pharmacies and sourced a month's supply for us, then we're able to get the next prescription themselves

"Our pharmacist goes the extra mile to source medication. We have to get separate prescriptions to try and source the medications from other pharmacies

"On occasions they have been supportive, but sometimes I have had to go to another pharmacy.

"Offered to release prescription to try and get meds from another pharmacy

"Not particularly, just said to try other pharmacies 14

"Not initially but did the reach out to local pharmacies to obtain from their stock

Just tell me to ring round

Just suggested I try other pharmacies in the area.

Content analysis of pharmacy support S2

Just been told they are on back order or need to speak to doctors to request a different prescription to try elsewhere (which we have had to do)

Just advise to use different pharmacy

In the past I had an issue with obtaining my previous Epilepsy medication, Carbamazepine. Often I would have to wait for days for the pharmacy to try to obtain some, twice I had to go to an alternative chemist.

I was told to just call other pharmacy's to see which of those had my medication in stock

I switched my pharmacy & they are always helpful & wonderful. It is a completely different experience.

I needed a spare bottle for holidays and the pharmacy messed up and left me 2 days to find some elsewhere myself because it wasnt enough time for them to fix their mistake. they said try this that and the other stores to see if they have it, i asked and if not to be given a shrug then i asked if it was possible to go to hospital for an emergency bottle as a last resort again to be shown a shrug.

I have changed pharmacist. On more than one occasion my parents phoned 15 + pharmacies whilst I was at work to track down meds and have travelled over 30 mins to collect.

I have changed my pharmacy & have had no issues with the new pharmacist either

I get told they don'thave any and to look elsewhere. I also get told it's a problem in the uk right now and to go back to the doctor. (I have already done this several times)

Helping to source from other pharmacy

Has reordered everyday, has called other pharmacist in the local area.

Content analysis of pharmacy support S2

Had to look around other pharmacies

Had a text saying they were unavailable and Staff in my usual pharmacy suggested I go to the pharmacy where I eventually got them.

Given me a prescription to go to other pharmacies to try to get it

Gave me the prescription telling me to try somewhere else

Don't think at my pharmacy (at the doctors) understood the worry, she just said I'll get the doctor to sign the form and you can just get it somewhere else! She also didn'tseem to be aware of the shortage

By putting the prescription back on the system so we can search other chemists in other areas who have it.

Basically said they hadn't got any Lamotrigine and told me to try ringing around other pharmacists

Advised to ring around to see if anyone finds stock

Advised me to contact Boots Pharmacy as they might have had access to wider stock.

Name: delays

variable. hospital pharmacist invaluable . i have patients ringing every week about difficulty with accessing AEDs. CBZ, Clob being very frequent recently. it takes a fair amount of time

My son was diagnosed in March 2023 with epilepsy I had to try over 20 chemists to get hold of the meds.   We have since had issues and have to order at least 10 days in advance

3. Late dispensing and late arrival.

Many times I have had to wait for my relevant medication to arrive at my pharmacy and occasionally I have come close to running out of anticonvulsants as a result.

Last month I asked the pharmacist to ring the helpline number as stated on the Epilepsy society website to request my medication but he refused saying it wasn't down to him. I got back onto my Gp surgery who happen to have an in house pharmacist that works there and he couldn't have been more helpful and chased the said pharmacist and with in 48 hours I had my medication. Not looking forward to collecting my next script and having to go through all this again.

Lamictal 25mg is the one I have experienced delays with. However as I order around a week ahead, any missing medication has been available before it has been needed

Keppra has been changed to weekly dispensing with no surplus supply

Just told me to wait, rather than let me know at the time they could not get.

Just told I'd have to wait

Just say they don't have it and come back another day.

Just said they would reorder

I was advised I would get a phone call when the medication had arrived. It hasn't yet.

I put in my medication request as per usual. However, after 2 weeks I had to chase the location of them. I had not been notified there was an issue, thankfully I had a supply of 200mg tablets so was able to bridge the gap. Unfortunately, not everyone is able to do that therefore they could have been having seizures. I felt unseen as someone with a condition, I felt disregarded.

Content analysis of pharmacy support S2

I have to go in and a couple of times as they say there not delivered yet. It is a long way from home.

Has always ensured I have enough to cover until remainder expected, and outlines expected delay.

Gave me what they had (enough for two weeks) had spent a week without before getting them.

Name: early request of meds

When the pharmaceutical company confirms stock will be back, I have had to contact my pharmacy to remind them to place an order. The date of delivery is also always extended.

Usually prescribed two months at a time. The pharmacy had one month's supply and re-ordered the second month which was dispensed a couple of weeks later. Rasagiline. No issues with the other medication

Tried each day to reorder

Told me to ensure to order earlier than usual. Local pharmacist assured me as soon as stock available, she orders enough (may have just tried to make me not worry?) Managed to get what we needed, did have to have 25mg Lamotrigine instead of 50mg at one point.

My pharmacist has advised me to request my medication earlier than usual

I was told when I first prescribe to allow a 1 week notice but they have always fulfilled my requirements.

I order 7 - 10 days before the medications expire and the pharmacy have them ready in plenty of time.

I have to reorder my repeat meds every two weeks but my GP won't ok early repeat

I always re-order whilst I have one month supply left. Our GP surgery and local pharmacy state a 7 day fulfillment of prescriptions. This time it took approx 3 weeks.

Fortunately I am allowed to order my medication 10 days before the issue date which gives the Pharmisist plenty of time to get the tablets

Always a month in advance currently for me

Name: extra support

Yes but I don'tdrive and work 9-5 M-F job (they started delivering the medication)

Name: GP not prescribing brand

Pharmacy was unable to source generic version of medication. Pharmacy had branded version, Lamictal, in stock but could not dispense as branded version not specified by GP. Pharmacy urged GPI to allow them to dispense Lamictal but GP refused. 1 day before supply of medication ran out, I located other pharmacies that had generic versions available. Eventually secured medication - 3 different generic brands for 3 different doses - a very stressful experience. Pharmacy tried very hard to get GP to allow them to dispense branded version, Lamictal, but GP refused.

My usual pharmacist did not offer a replacement dosage. For context, I use 100mg capsules of Zonegran which currently have a shortage issue everytime I get my repeat prescription. The pharmacist did not think to offer 50mgs as an alternative, which I find are usually in stock. It was only after trying a second pharmacist that they said that would be an option, but would require a new prescription being drawn to request the exact 50mg dosages.

It was my usual surgery which was obstructive, first not supplying Tegretol along with the rest of the meds on my repeat prescription, then telling me that I was not due to have them even though all my drugs are supplied together so are closely synchronised. My usual

pharmacy did not have them but did at least inform me of the shortage, give advice, and succeed in obtaining them for me in the nick of time.

Name: independent pharmacies helpful

Yes and no. Boots are where prescriptions automatically go to and they have not been helpful. I've rang around 10s of others and have found 1 helpful independent pharmacy who understands

We use an Independent pharmacy

Usual pharmacist suggested trying an independent pharmacy, eventually found one who had some in stock

Two pharmacies made it clear they were only willing to try and order it once a week as they were at capacity, even when this risked him running out completely. In the end a friend with the same medication has tracked it down at an independent pharmacy and informed me. This pharmacy has been a lot more supportive and helpful

They weren't particularly helpful just told me that they would release the prescription and I would have to call around every pharmacy until I found one that might have them. This is not a generic brand and they said they had run out. Fortunately, I found a small independent who were great and said they would help me and call me once they had located some. I now order 3 so I know I will always have them.

"They only have so much in stock Community pharmacists seem to have buried their heads in the sand Won't easily allow patients to switch from CBZ P/R to I/R without something in writing from consultant to cover their backsides

"Only the local independent pharmacy has been helpful the larger stores like boots have been very dismissive

I have had to change pharmacy and although the smaller independent pharmarcys seem more likely to try and assist following up getting them I've had to switch to a larger

pharmacy because Im currently on a newer medication and difficulties with it being in stock cos of of costs to them I think. They are also not ever available on the phones at Boots pharmarcy when they say they will be so you just cant get hold of anyone.

GP surgery pharmacist messaged me to say local supply issues, then she called my community pharmacist to confirm availability there (in another area) and he was able to source it

Doctor Pharmacist was great and explained it's best to go to independent pharmacies as they are not bound to national contracts.

Name: insurance issues

"Pharmacist was great but insurance was denying payment

Name: medication out of stock

We can't get hold of the Accord brand of lamotrigine. The pharmacist has said that the big companies like Boots get the pick of the medicines and whatever is left goes to the smaller pharmacies.

Very apologetic and they keep trying to get them!

Usual Pharmacist just said 5mg lamotrigine is out of stock and contact my daughters paediatrician. Other pharmacists have varied, most won't help, one did manage to order some after contacting different wholesalers for me

Tried to find them but am difficulty.

Tried different suppliers

Content analysis of pharmacy support S2

They've tried and are apologetic, but can't reliably fix it

They've continued to attempt to get slow release tegretol and phoned other pharmacies to check availability

They try. Not always able to get her onfi

They rang around and. Keep my prescription open until stock was available anywhere in the local area.

"They just say that they will inform me when the medication is back in stock1

"They just lie to saying they have some to get be by and the next day said they said the have none and they do know when they will be get any in1

"They just keep trying but have to order upto 14 days in advance

"They have given my husband a weeks at a time, he's attempted to collect medication several times a week and it's not there/ not ready/ not recorded as required. Many times he's been questioned on if he has had his months worth but he hasn't. They aren't proactive

"They had a shortage on the specific brand I needed. I actually had an allergic reaction to the GSK Lamictal brand which caused rashes on my face and rashes and sores on my lips I was soo unhappy and Self-conscious.

"They are trying their best to get my medication.

"They apologised at 4 of my local chemists, and stated they had no idea of when it would be available. I had to cut my dosage by 50% for a week. Luckily I returned to a local chemist and carbamazepine was available.

Content analysis of pharmacy support S2

"The pharmacy has been unwilling to order Tegretol retard directly from Novataris,the producer. They merely kept telling me there was no Tegretol retard, which was/is not the case.I found the GP pharmacy helpful in sourcing some from a pharmacy further away. It took for me to write to the pharmacy telling them I was writing to my MP for them to order directly

"The pharmacist has tried all his supplies to ensure we have had the Lamotrigine we require. We have had no problems with the supply of Keppra

"Tesco pharmacy said they couldn’t even order the Rasagaline

"Tegretol know there are national shortages they do not understand or give updates between my visits to them . Keppra they do not always order right brand and understand importance of doing so. Depends who is doing it.

"supportive, but to no avail as there was no import into the uk for zonisamide for a while and also Frisium last year for a while

"Sort of, they contacted their suppliers only to find out none of them had it.

"Some have been great, but some have struggled with finding solutions as they are a large chain.

"She has contacted 56 pharmacies as they are having difficulties due to suppliers. They gave me 8 tablets of 5mg as apparently that's all they had so it was an opened box that should of had 36 tablets in it. I explained my daughter is on an increase plan up to 90mg so she needs 3x5mg every night then from next week 6 x5 mg tablets per day along with her 25mg to make up to 90mg which we thankfully have. Now from tonight I have had to move back down to 75mg because I don't have anymore 5mg tablets to give her the increased dose of 90mg. Tomorrow is Easter Monday so I am not sure when I will hear back from epilepsy nurse and the chemist couldn't give me anymore advice just that 'hopefully she will move up to 100mg next' as she takes 3Ã— 25 mg and then the 5mg. Although I know she won't go up to 100mg it will be 105mg as she increases up in 15's not 20's.

Content analysis of pharmacy support S2

"Several pharmacies have checked with their suppliers and all gave the same response, that the product is out of stock nationwide.

"Reassurance but no guarantees

"Rang different suppliers

"Put on tegratol and now shortages getting random amount of days medication

"Pharmacy are very short with you and unhelpful. They don’twant to understand that it’s not something you can go without. They say they’ll try and order it to later find out they did not. You keep going back because you can’tgo without and they get annoyed at you

"Pharmacy app but lately taking longer to arrive cos they out of stock

"Pharmacy claims to be always waiting on the delivery. I had to reduce the dosage of Pramipexole while waiting

"Pharmacist unable to source either 200mg or 400mg

Pharmacist is just telling us that there is a nationwide problem making us worry more

"Pharmacist has suggested alternative medication brands when available.

Pharmacist has gone above and beyond,even as far as bulk ordering a month supply in advance. They were recently bought out by a chain, rang to let me.lnow they could no longer support well in advance,. New pharmacy have managed to supply 4 months supply of new med for now and have been helpful in getting meds other meds that are in short supply.

"Pharmacist has been trying to find out when things come in or where else available

Content analysis of pharmacy support S2

"Pharmacist can't do anything if it's a supply problem with the manufacturer

"Pharmacist 18 months ago in Lloyds Pharmacy was very poor, and I switched, The pharamcy has closed since.

"Order via Lloyds, Just wait for it to come in stock amd they send as and when the different MGs come in. Order well in advance so never short

"No, just told me they were not available

No our chemist had the 50mg in stock of briviact and refused to give that as an alternative. This would leave my daughter run out of briviact.

My pharmacy informed me oxcarbazepine is completely out of stock at all their suppliers but they will keep trying to order it in. I had the same problem last time I went to collect my prescription.

My independent pharmacist, was the one who told me my medication was not available and said that the doctors had known for some while. He advised me not to take the medication the doctors had told me to take as one was not pronlonged released as I take and may cause issues. So I contacted the Epilepsy nurse at the hospital how was able to contact the pharmacy and help me.

My husband went to collect my prescription as I was in work. He was handed my prescription and told they couldn't fulfill it. They had had it for 10 days. No warning.

My husband was left short of his rasagiline , there was no dialogue, only an email

Just told to come back, "its out of stock" I was off for a week so went in every day. I only found out there was an Epanutin shortage when I asked them to check. They were a waste of time really.

Content analysis of pharmacy support S2

Just tells us it's out of stock

Just tell us there’s a shortage. Don’toffer any other solutions or ring any other pharmacy

Just said they would reorder

Just said they can’tget it and will tell me when they can

I've been luck and the pharmacy has been very supportive phoning the suppliers directly on my behalf. I've taken and prescription to over pharmacy's to but they are now struggling to get Tegretol prolonged release

It is more a case of shortage of pharrmacies as a chain closed in our area so now have up to 5 days wait for the request to leave the surgery and up to a further 10 days to get through the pharmacy.

In January this year, a week before my regular medication renewal was due, I was forced to go on a recce of local Pharmacies in search of both my Tegretol and Clonazepam (at the time restricted to Auden by TEVA UK Ltd) medications as my normal supplier had no stock. My usual Pharmacy had only Generic 500mcg Clonazepam tablets but more worryingly had no stock of 200mg Tegretol (or other dosages of the same) and did not know when they would see any. I eventually found one Pharmacy out of four in the area that had stock of both branded medications at the correct dosages but understandably would not set aside any for me without a valid prescription. I had enough Clonazepam to last me through the first week of February and sufficient Tegretol until mid February. Thanks to quick action by my local Health Centre I was able to revisit the same Pharmacy the next day to collect the Tegretol but unsurprisingly their stock had been depleted in the meantime. As regards the Clonazepam they were only able to offer a months supply of a generic version and not the branded type as above. The Pharmacist duly refunded a month's supply of each on the dispensing token to allow me to source a supply elsewhere. That supply I eventually located in a Pharmacy 40 miles from where I live. It has been a monthly endeavour ever since to find a Pharmacy that has been able to provide the cat 1 ASM that I require after having been on the same branded tablets for over 40 years. As regards the cat 2 helper ASM (Clonazepam) the resident Pharmacist at my Health Centre, in discussion with my Doctor, has applied a clinical judgement and switched me to a generic brand to ease the burden of supply. This unprecedented situation has caused me immense anxiety over the last few

months and continues to do so as I hunt down a supply when every medication renewal falls due.

I'm on a new drug, Ontozry, while reducing the Lamictal, so my epilepsy which isn't fully controlled is in a potentially dangerous situation. My chemist can't always get the new drug on time but he does fully support me. Last week he said that first there wern't gps available to sign my weekly prescrition , he had to go three times to my surgery. When he got it the wholesalers here in Belfast didn't have Ontozry and had to order from England. He did mange to get them just before closing, but it's been leaving me anxiuos

I've been told by pharmacists that there is no medication available

I'm wheelchair bound so it's a trek to any pharmacy and the answer they give is â€˜try again'â€¦.(whatever date)

I'm not sure, perhaps yes but the medicine needed was not available so they asked me to go to another pharmacy due to no medicine the day I needed it. When I couldn't find it at two other pharmacies, I had to come back to my usual pharmacy and ask them to just give me my other medicine please, though they said they had cancelled it & didn'thave the right amount for me. (Instead, I had taken 25mg more for 3days whilst lowering a dose, then later going back down to my usual when receiving my correct amount from the pharmacy). Within this time I had suffered, perhaps 2-3 seizures within a week.

I use Lloyds Direct and they just say that they are working to resolve the issue. I have come within 1 dose of running out of my 100mg twice daily

I had to direct them to the Epilepsy website with updates. I am constantly told next week but of course next week comes and no medication

I had 2 weeks supply one month, then had to hone anather pharmacy foe next 2 months supply, the one month of the non slow release drug.

I am told to find a pharmacist that stocks the medication.

I also contacted my epilepsy specialist nurse who recommended my surgery with alternative after I had contacted drugwatch about shortage and difficulty of tegretol prolonged release

He tried really hard but wasn't able to get it. Hospital have been, but can't get the next lot. Scared stiff for the week after next

He has tried to place an order but hasn’t done much other than that.

Have tried to get medication

For the last couple of years, the pharmacist has been great. The GP prescribing service, not so much. In the past, the pharmacist has moved mountains to access a medication (which is no longer taken) that was in short supply.

Explained why there was a shortage

Everyone has been trying to access it but there is no supply

But when I expressed concern re possible shortages, the dispensing team within my surgery did not seem aware of any shortages. They assured me they would not let me run out - but perhaps this could end up out of their control?

But unable to source it

Brief shortage. Managed to get some eventually however previous medications have been worse experiences

Between Feb 2023 and October, I was on different meds and quite often pharmacy were unable to source my medications including the lacosamide, they did try to communicate to other local pharmacies to even attempt a weeks supply, as an epileptic and not being able to drive to collect this added fuel to my anxiety and temperament. Before Christmas surgery would not prescribe 2 months of pills for me so I wouldn't have additional anxiety being around Christmas music and busy environments but since I have changed pharmacy and explained my difficulties my surgery and pharmacy have been very understanding in

allowing me to order my repeat prescriptions a week earlier to ensure availability and providing of product although each occasion I have had a different brand, however still can only can get one month at a time, it doesn't help if you are trying to plan a vacation.

All I keep getting told is â€˜ it’s out of stock’ no help whatsoever

Name: no alternative

We had a shortage of Tegretol slow release capsules (for my 8 year old son). The pharmacist told me she had declined our prescription from the doctors and that she couldn’t give anything as an alternate. No help on what to do / alternate pharmacy’s etc

Variable. Although previously very supportive we reached a point where our original pharmacist was essentially saying they could not help any further. We changed pharmacist and our current one (Boots Milford on Sea) has been very helpful

Usual Pharmacy, depends who you speak to. Other pharmacies vary from extremely helpful to not even wanting to try

Useless pharmacist. Just prescribes genetics which cause fits

Unknowledgable and unhelpful pharmacy staff, who have done nothing to help me source the medication we require adding more stress to an already stressful situation

They have not been telling me they can’tget hold of the medication until I turn up to collect it. I ask them what I should do and they say they do not know. Very unhelpful and due to this I have had to miss a few doses

"They don’tcare

"They didn’tseem to think it was important

Content analysis of pharmacy support S2

"The pharmacist will blame the Gp surgery then hang up the phone while I am mid sentence trying to explain, The GP surgery will blame the pharmacist then hang up while I am trying to explain mid sentence. This leaves me contacting NHS healthwatch, adulst social services safeguarding team etc. Then both the pharmacist and GP surgery lie and say they have not let my 84 yrs old very ill mother without her medication

The pharmacist just said they didn't have any and couldn't get any and sent me away.

"Supportive in words but not able to help in any practical way

"Suppliers don'thave it either, so what can they do?

"Pharmacist has been awful. No foresight as to when there may be deliveries. No waiting lists so I've had to rely on the help of my parents to constantly check if they've been delivered on the off chance they have some in stock. We've been able to get a box here and there. They still owe me hundreds of tablets, with no communication as to what might happen next.

"Our local Well pharmacy has a different pharmacist and counter staff every day. They have to work very hard in unfamiliar surroundings but can take little interest in the ongoing care if patients. They have no interest in accessing Topomax fir me.

"Only one alternative pharmacy was suggested, when asked about what other pharmacy I could go to they said I had to find out myself.

"One has been supportive but two others said to try elsewhere with no suggestions of where, leaving patient stressed

"No support to look elsewhere. New pharmacist, old pharmacist went above and beyond. Son's prescription goes to one side as he has a number of medications

No medication in stock pharmacy don't offer any other brands or offer an alternative dose to double up

Never offered to try other dispensary.

My pharmacist is unable to obtain Rasagiline and gave me no advice on how to obtain it.

My current pharmacy (Boots) have been helpful, however when I was at Pharmacy 2u, then they were exceptionally unhelpful. It was impossible to contact them about anything and the item just didn't turn up, leaving me with no item. In the end I cancelled with them because it was too unreliable. I'm still getting messages asking why I left them...

I was told they couldn’t get it and that was that

I was just given the slip of paper they give they always give when short on a drug. No further support

I use an online pharmacy and they are not helpful trying to find a supply.

He couldn't get it so just cancelled my prescription without getting in touch with me.

Basically told me they couldn't get it and offered no help or advice, I had to contact my consultant for support

Name: no concern

We are in a support living setting and have all medication supplied on a 28 day cycle and about 3/4 day before we are ment to get new cycle we are told they have no medication and still trying to get it. We then have to go back to Dr for new prescription to start ring round other pharmacy in and out of area to see if they have some.

My son lives in supported living, so I don’tdeal with his medication.

It only took a few days to fill my prescription

Content analysis of pharmacy support S2

I use pharmacy to you, order it monthly and have not had any issues with medication availability

I have occasionally had to contact them when supplies have become very low *Pharmacy2U online & I have always received supplies in time

I have my medication sent through the post from my online pharmacist and if they are having difficulty they try and give an e-prescription so i will be able to get it from a pharmacy near me which has been successful.

I have been given my medication every time I went to get them n if I fell short I only have to call the doctors and they give me them.no problem

Had no issues

Boots pharmacy are very good and work really hard to source supplies and ensure I have medication

Name: not a priority to them

They argue that from their perspective stocking my medication is too expensive and they'll keep checking to see if they can get hold of it for me but from my perspective it feels like I'm not a priority when it appears to be out of stock, I am often trying 4 pharmacies per month with the stress that I may end up in hospital

"They are very busy and it is not as important to them as it to me obviously

"The chemist has not shown any interest I am just told I will get a call when they can get it back in stock

"Sometimes they are helpful but then other times they are not

"Only helped when I got distressed about the medication not being available. The pharmacy didn’treally appreciate that this was a dangerous situation.

My pharmacist did not seem at all concerned and I had to insist he tried again to order more. He managed to get half of what I was owed and after a further two phones calls got the remaining tablets. I had to have a conversation in a crowded chemist about the risk of having a fit. I do not mind talking about my epilepsy publicly but some people are not. There was no attempt to give me any privacy during the conversation.

I felt really let down, they acted as if it was just another medication out of stock and didn’tunderstand or care how vital it was for me to have my medication.

Have to get doctors to tell them why I need medication on time

Name: out of date medicine

"Sort of, my husband has been given out of date (within a month) medication.

Name: providing additional support

When my latest prescription was due, the pharmacist gave me one box of Tegretol until stocks become more freely available.

Very understanding. Now delivers meds to me to ensure no problems.

Very supportive. Always tries various suppliers and have even started trying to source my next months supply early so if there is a delay it’s hopefully resolved before I actually need the meds.

Content analysis of pharmacy support S2

"They suggested generic but I explained it needs to more precise. She tried to help by suggesting a pharmacy that might have it in stock

They send what they have and get from other chemists in their group

They seem to have a â€˜emergency stockâ€

They rang around and. Keep my prescription open until stock was available anywhere in the local area.

"They have tried to get the same brand and been unsuccessful

"They gave me a week's worth to tide me over and got the rest asap.

They gave me a couple of strips to keep me going until they could fill the prescription in full. I asked whether I could try and get it from somewhere else. I was told as they had partially fulfilled it, I couldn't go elsewhere. I was not aware of this. I had to keep calling to check whether it was in

"They called round all chemists they could to get it for me and delivered it later on that day when they got it

"The pharmacist has always given me an emergency supply,of about 4 days

"Some have some haven’t. Many say they are too big to be able to â€˜go off piste’ and try to source from other areas. Virtually no independent pharmacies round here so I resorted to the hospitals’ pharmacies who were brilliant.

Pharmacy really supportive after a lengthy conversation. Previous to this I was told it doesn't matter unless you have epilepsy... And having to answer actually I do.

"Pharmacy has be trying to get them in for me

Content analysis of pharmacy support S2

"Pharmacist in Boots very helpful and ended up pointing us to an alternative supplier.

"Pharamist is very supportive and seeks all methods to source the right brand as I'm brand specific. She has even cut tablets in 2 to make up the right dosage for me

"Our present pharmacy has been very helpful trying to supply my correct brand of medication. Lot of others have not, some seeming disinterested in trying to help at all!

"Our pharmacist is brilliant

"Offered me 100mg which were dispensed after a new prescription raised.

My pharmacy have been supporting and tried their hardest but my local doctors not as much

My pharmacy are very upfront and helpful

My pharmacist was brilliant at getting it for me

My pharmacist is Automeds.co.uk who collect my prescription from my doctor's surgery, divide the medication into trays of capsules & deliver them free to my door.

My Pharmacist is always notified in advance by at least two weeks, of my need's.

My Pharmacist has been trying ringing round other Boots for me , is kind and understanding.

My online pharmacist contacted my doctor for shortage of one dosage to arrange an alternative.

use my local pharmacist Weaverham Pharmacy in Northwich and the female pharmacist has been the most supportive medical professional I have ever come across. She has advocated for me and ensured I have at least 4 months stock of medication in advance to

ease my concern and to combat the shortage as much as possible. Her communication is incredible.

Just given enough to keep going and told I need to give as much notice as possible as it’s hard to get hold of

It was because of the pharmaceutical companies that I new there were trouble getting the tablets no body else informed me and when I rang around if they had some they would keep what they had under my name so I could get my drs to forward a skript over for what they had this has been very helpful.

I'm a very sensitive to variations and my pharmacist has to ring round his suppliers to get the right keppra manufacturer as since Brexit he has been having difficulties in accessing it from his usual supplier (ie the outfit that fills his prescriptions, hope that makes sense.)

If they cannot get the medication they just say â€˜you will have to try and get it from another pharmacy and leave me to search other pharmacy’s

If the pharmacy didn't have it they will help locate a place that will have it at the time

I’ve ordered earlier and they’ve contacted me to let me know that 1 of the medications wouldn’t be in with the others and would be delayed. Luckily I had days left over due to a shorter month but I was glad they was able to help me out

I was given different brand of medication once, however, when I have raises the issue our pharmacist was very helpful and ordered the brand I usually take

he's contacted diffrent suppliers, managed to get me a months supply of tegretol

He has been fantastic. When he can get the zonisamide he has been getting 3 months worth to ensure he has it as stock are not available. I know this sounds selfish and doesn’t help the situation.

Boots tried their hardest they rang other pharmacies keeping me posted

Boots in Milford on sea have been very good and called around other pharmacists to find what i need

Name: providing available supply and owing

Yes my pharmacist has been proactive in finding my medication. As soo as they knew there was going to be a shortage they ordered my next months supply and kept some aside in my tray to last me until the end of the shortage

Yes and know. My previous pharmacist chemists for u just send an incomplete prescription without warning. Because of this I changed to a high street pharmacy who tell me if the prescription is short and reorders. This can take up to a week. It is only my PD meds that are out of stock. This happens every month. My

When there is a shortage, the pharmacist will make up however many weekly boxes he can, it's then delivered and there is a message left so that I know what's happening.

When I gave in a green prescription for tegretolf prolonged release 200mg tabs and 400mg tabs for my son. They just gave an owing slip for the 200mgs as they had none in stock. When asked when they would be back in, they said they didn't know. It was over a month before they had 200mgs back in stock. When I found out they had none in stock, I got in touch with my paediatrician to say they were out of stock. The paediatrician then organised for me to collect 200mg tabs from the hospital pharmacy.

We were told by the neurologist at UCLH to make sure we stick to the same brands. That's been impossible !!! I've had to ring around different pharmacies to find the meds but in the end we just have to have what they've got and the prescription is often incomplete and gave been given an owing slip

They'll give me whatever tablets they can and then give me a pharmacy sticker indicating how many tablets I still need and tell me to take it to other pharmacies to see what they have

"They usually allow a pick up of the missing meds a few days later BUT I no longer drive due to recent seizures so picking up meds once is difficult enough without having to arrange a driver for a second time

"They send what they have and get from other chemists in their group

"They provided part supply or sent reminder to manufacturer. However I am not informed by the pharmacy if there is a problem obtaining the medication until I go in to collect.

"They have given me what they had and then given me an I.O.U slip for what was remaining for me to receive when it next came in to the pharmacy.

"They have given me part of a prescription and later the rest.

They have either told me they only have a small amount available but gave me an owing note and to check back in a couple of days when the next delivery arrives. It’s very rare that I have to go to a different pharmacy to get my medication

"The pharmacist issued me with what he had but couldn’t fill my full prescription

"The pharmacist in our village (Boots) is generally good, but if they can only get some of the medication we are obliged to go back to the GP for another prescription for the parts they cannot get. This is time consuming and very stressful. Luckily I am fit, assertive, and can drive. My daughter would find it impossible to chase these medications otherwise. Poor communication between GP and pharmacy now - it used to be good

"Sometimes pharmacist hasn't got it or my main concern is that I an constantly short changed. GP prescribes 100 tabs but pharmacist issues 90. Have told GP, nothing done. Pharmacist says he has to issue boxes of 30 so I get 10 less every month

"Sometimes not all amount is available immediately

"Provided catch up prescriptions for missing medications

"Primidone temporarily unavailable but he gave me what he had which lasted long enough for the Primidone to come back in stock.

Content analysis of pharmacy support S2

"PHARMACY USUALLY OFFERS ENOUGH MEDICATION TO LAST ABOUT ONE WEEK WHILST THEY GET MORE

"Pharmacy guy gives me less medicine,for 2 weeks and we hv to travel 20km to reach him

"Pharmacy 4 U have been terrible on 2 ocassions where part of the prescription has been out of stock. They've even sent the wrong notification on their app to state that it was fully fulfilled so I wasn't made aware until receiving part delivery

Partially, they have provided no visibility on when the Tegretol will be available, at one point laughed bc I was stressed at them giving me only 1-2 weeks supply.

"Our local pharmacy has mined to course 2 bottles for our monthly script and will continue to do so but give me plenty of time to source elsewhere if he can’tfind it

"Only being prescribed enough for 1 month. Am at the gps or chemist at least twice a week. Always partially filled prescription are available

"Often only get half of my medication and have to go back for the rest another time

"Normally have my full monthly medication but whenever has some shortage then chemist will give me some for two and asked to come back for the rest of prescription.

Many have checked warehouse stocks and issued part-precription if any on shelves

Just handed partial prescription and told 'we owe you' no firm date of when they would be there

it is frustrating that i almost always get an owing note for my Epilim and have to keep phoning the pharmacy up asking if they have got the rest of my Epilim

I was only given half my month's supply of Keppra

Content analysis of pharmacy support S2

I rang epilepsy action and they gave me details, I had to contact pharmacy and give instructions. They could still only.offer half my prescription

I get an initial amount when I put the order in but usually the order is short so I get half and have to go back to Chemist a week later for the rest

Have had shortages of Lamotrigine which they have had to owe me

Gives what they can

Given me what is available until the pharmacist has the rest of the amount

Every time I've gone to collect my regular prescription of tegratol, my chemist has not had the full amount, they always have to owe me a box. most recently, they left me for a week, I had to go searching and asking every local chemist, including boots who could only give me 6 tablets, , I was left with 2 days of medication,, they suggested I ask for a alternative, which no one had suggested before, I had to go back to gp, ask for a new prescription, back the next day to go to collect prescription, for tegratol 100 mg, I am now on this, I am experiencing side affects of this ,which are pins + needles all over my body, this is half my dose!! , I have since found a local chemist who is trying to get me the 200 mg tablets, I have spent most of the week talking to gp, discussing how best to manage my dose of 100mg tablets to replace the prolonged release, I don'twant to continue switching medication permanently, I asked my usual chemist to try and order my medication from Novartis website, but they said they can'tbecause they are part of well pharmacy chain, and they have their own supplier. Please help , and advise what is the long term solution, this is very stressful, and stress triggers my seizures.

But they normally only get half of it or less and then we have to try and get the rest later

Been a struggle many times I have had to collect sometimes a box at a time and make extra trips to the pharmacy over a week in advance to ensure they get some in before I run out

4 visits to obtain 1 full script with pharmacist trying to get delivery

Content analysis of pharmacy support S2

Name: sent back to GP foe new prescription

When I asked the pharmacist what I should do, instead of helping me and directing me to another pharmacy they told me to just ring my gp. This was incredibly distressing and I had to go to work, I spent all morning crying and phoning pharmacies in the county trying to find somewhere that had my medication, I was then back and forth with the gp trying to get a code to pick up the medication from a pharmacy which took all day. I spent the entire day worrying that I was going to have a seizure in the night as I had no medication left

We were told to go back to the GP to have a different medication prescribed.

we are just sent back to the Dr to re issue a prescription for a different dose

Was advised out of stock but will chase supplier; speak to your GP

Tried to get Rasagiline from other branch but I had to get new. Prescription from gp and go to another town to get it filled.

To begin with , they said the wholesaler had no tegretol. When I flagged that this would have an impact on my seizures they advised I go to my GP and get a different medication or carbamazepine. I contacted Novartis who stated they could provide Novartis but needed the pharmacy to contact them direct. I informed the pharmacy (Boots) but they said they as a local pharmacy could not contact them as they were a national company. I contacted Boots customer service who put me back through to my local pharmacy. Boots have now gathered half my prescription so I won't run out immediately but this concerns me.

"they suggested I should talk to my doctor to get an alternative prescription (i.e. ordinary release tegretol)

"They offer generic brands, then will only hold prescription for a few weeks until they cancel it then you have to get a new one and explain to doctor what happened to the other one and then start the whole process again

Content analysis of pharmacy support S2

"They notified me when collecting my medication that they didn't have the brand of lamotrigine that I use in one of the doses but was able to get in touch with the GP to resend another prescription with the lower dose tablets.

"They just tell me to go back to the my GP I'm just a number to them

"They have told me to contact my gp for different medications, but I know they don't work11

"They have just said to try another pharmacy. As the prescription involved more than one drug, this meant going back to the GP to get a separate prescription

"They have advised me to contact the GP to arrange an alternative

"The pharmacist just advised me to go back to GP and get another prescription so I could shop around. I had to get taxi to a number of Pharmacies as I find these chemist never answer the phone. I did manage to get a weeks supply of the 200mg I them called epilepsy nurse at QE Hospital in Birmingham she has sent 2 weeks of 200mg I have the 100mg but it means giving Mark 6 x 100mg. I hope I don't have the same trouble in a weeks time

"Supportive, but I was not informed of any shortage till the last minute by the pharmacy, my request to the GP for an alternative dose (100mg as opposed to 200mg) was then very urgent. The GP surgery response was extremely poor and very lengthy

"Suggested I get the doctor prescribed something else. When told was not possible, told to break 400mg tablets in half to get the required 600mg needed twice a day

"Sometimes, but depending who is on duty they will ask you to get your doctor to re-issue prescription so you can go elsewhere which is not a viable option due to boots chemist closures in our area and no transport (sister in tilt in space wheelchair, so need wheelchair taxi which are hard to get hold of and the council stopping numberous buses in our area). Always have problems with getting hold of my sister's medication even if there is not an actual national shortage. Have in past had to contact the manufacturers of medications to find out where I can get some. A lot of the time it seems to be the suppliers who chemists deal with who promise it will be in stock then it not turn up. To answer question 4, have had to ring around chemists in the city of Nottingham where we live.

Content analysis of pharmacy support S2

"Sometimes more issues changing prescription from gp to available medication

"Sent me back to the Dr and left me to ring around for the medication

"Sent me back to doctor

"Said their manufacturer couldn't get it so my daughters neurologist had to help

"Returning prescription and suggesting I try other pharmacies

"real answer 'no' but then 'yes'... A pharmacist suggested I go My GP for an alternative, I felt neurologist probably best. Left numerous messages with newish PD nurse/neuro office/secretary - nobody got back to me. Whilst trying to decide next course of action the pharmacy messaged me to say they had found a supply. I'm still trying to contact someone to find out what I do the next time..

"Pharmacist has mainly instructed us that they cannot obtain and we need to seek alternative prescription from GP. We have then had to phone various pharmacies to establish what stock they have and then act on that.

"Only GP can change it so no.

"One time (2 years ago) my chemist gave me a partially full pack to keep me going, until the chemist received more stock

"Not really they tell you there is a shortage and send you back to the GP's surgery for another prescription to take to another chemist

I was told to return to the GP who would find another suitable medication.....which she did

I was told to ask my GP for a different medication!

Content analysis of pharmacy support S2

I am always told to go back to my GP to prescribe a different medication. When I explain I can't do that with my epilepsy meds they just tell me they cannot get that specific brand in stock and unable to give a date when they will have it.

Had to return to GP and then Neurologist to get alternative drug

Explained I should go to my GP and ask for a different dosage of tablets

Emailed GP to ask if they could prescribe double does and take half at a time. GP did not reply. Pharmacist asked me to chase GP. GP said yes. GP then said no and told me to go to another pharmacist a mile or so away. They were helpful but said that my normal pharmacy still had the prescription. They kindly phoned my normal pharmacy who then said they now had the correct tablets. At no stage did any party phone me with an update.

But unable to get rectal diazepam at all 10mg and had to send prescription back to go who did nothing

Although he didn'thave the same brand he did ring around other pharmacies to see if they had my brand and dosage. When they came back and said they were out of stock he advised me to speak to my gp and see if they could re-issue the prescription at different quantity so I could stay on the same brand

All we get is 'we don't have any, somewhere else might, ask your GP for another prescription as we have marked this one and we can't give it you back.

Advise me to talk/ message my dr to temporary change to the 100mg tablet

Name: struggle with hospital pharmacy

> Yes and no. I have to get half of my medicine from my doctors and half from the hospital the doctors pharmacy are good it's the hospital whom I struggle with

Name: supply issues

Yes, but now the supplier can’t supply anymore 10mg diazepam. This is a big problem as it’s an emergency medication for me

"Pharmacist says out of stock/cannot get from supplier
