# Supplementary material for: Experiences Reported by People with Epilepsy During Antiseizure Medication Shortages in the UK: A Cross-Sectional Survey
Source: Pharmacy (Basel). 2025 Nov 10;13(6):166. doi: 10.3390/pharmacy13060166 (PMC12641818; doi:10.3390/pharmacy13060166)
Supplement: Supplementary file 1 [file pharmacy-13-00166-s001.zip › Gender distribution for Epilepsy Action s5.pdf]

Gender distribution for Epilepsy Action

Gender	Percentage (%)
Male	17
Female	35
Prefer not to say	48
Other	>1

Note: Percentages have been rounded off to the nearest whole number.
