# Supplementary material for: Experiences Reported by People with Epilepsy During Antiseizure Medication Shortages in the UK: A Cross-Sectional Survey
Source: Pharmacy (Basel). 2025 Nov 10;13(6):166. doi: 10.3390/pharmacy13060166 (PMC12641818; doi:10.3390/pharmacy13060166)
Supplement: Supplementary file 1 [file pharmacy-13-00166-s001.zip › questionnaire s4.pdf]

Questionnaire of the study

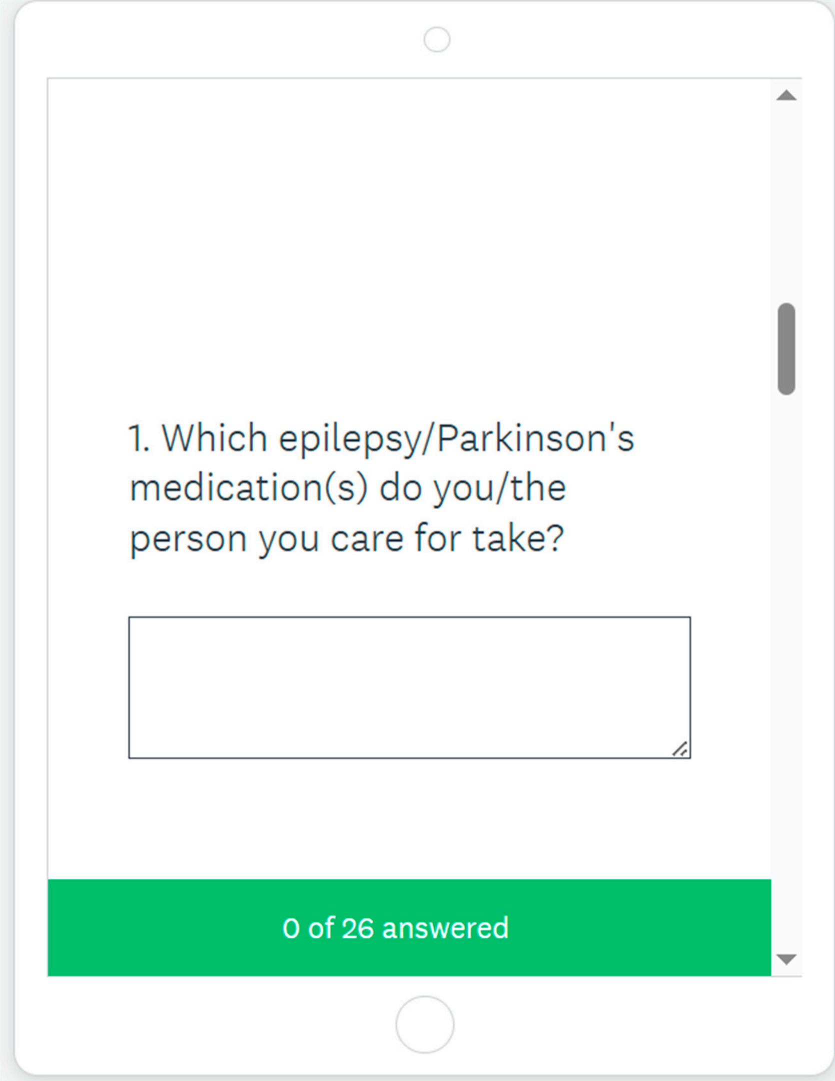

Questionnaire of the study

2. Have you/the person you care for had any difficulty getting your medication in the last......?

- [ ] month
- [ ] three months
- [ ] six months
- [ ] year
- [ ] no difficulty

Other (please specify)

0 of 26 answered

Questionnaire of the study

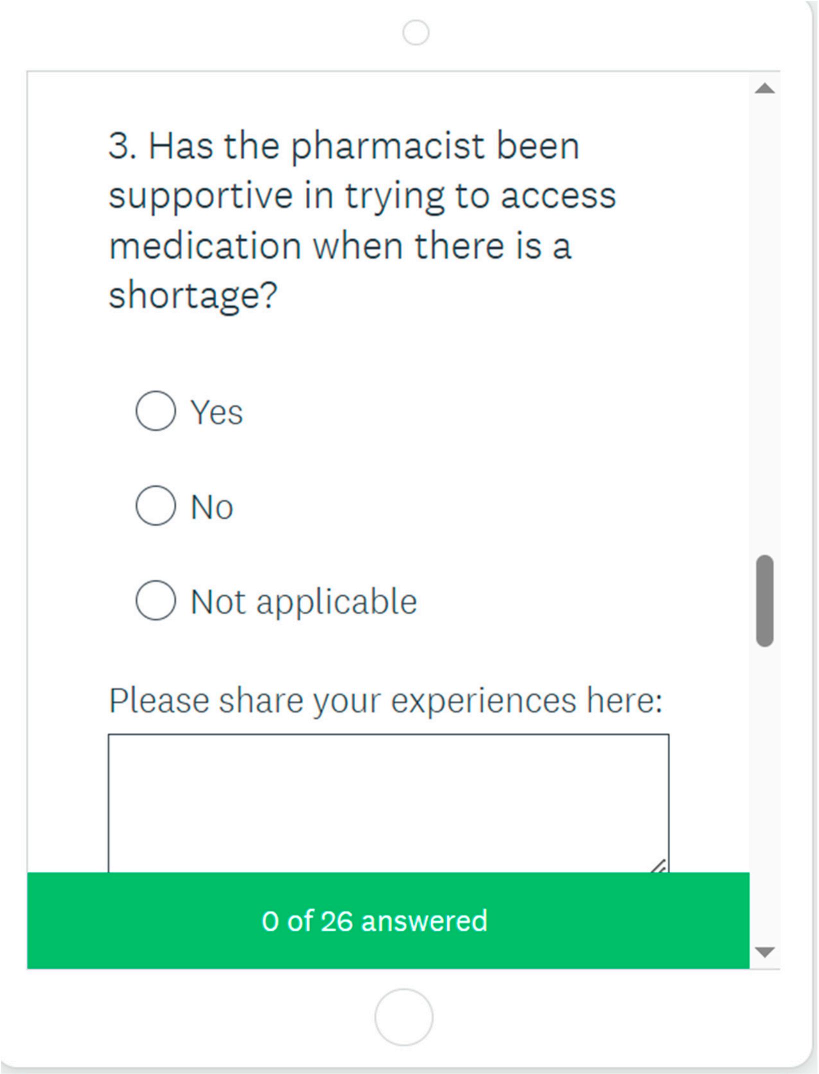

Questionnaire of the study

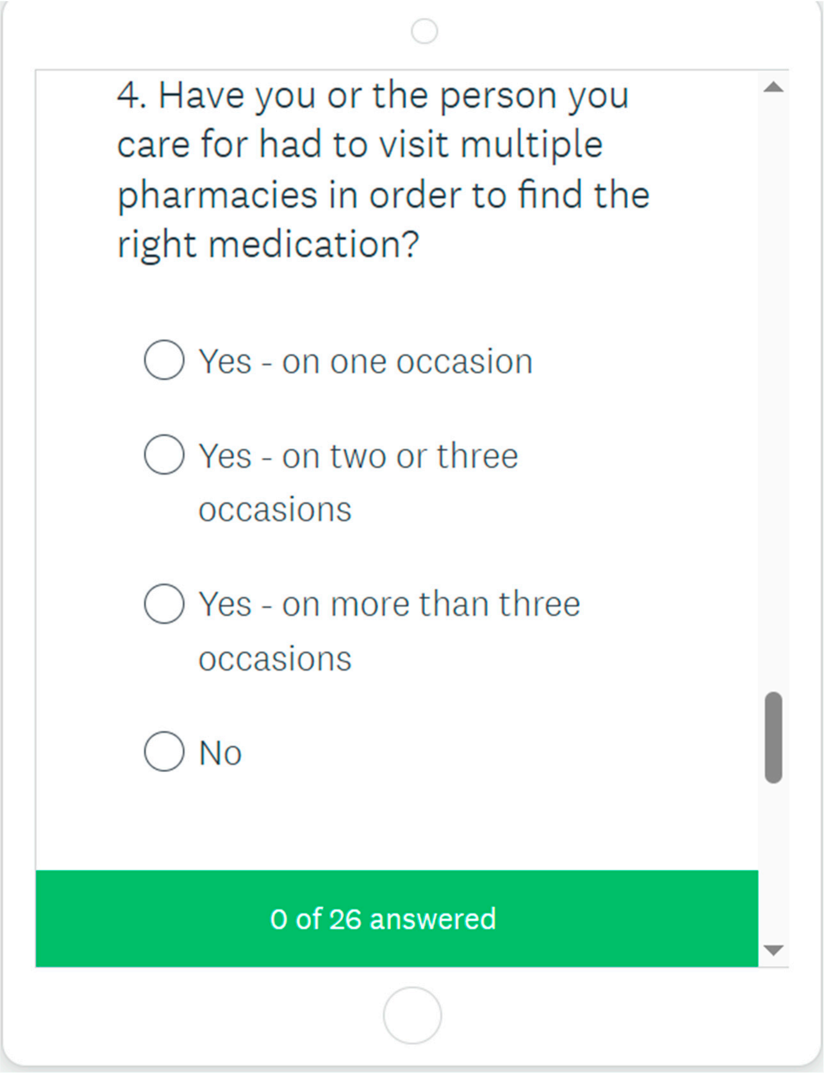

Questionnaire of the study

5. How many pharmacies have been visited before finding medication?

- Just 1
- 2 to 5
- 6 to 9
- 10 or more

0 of 26 answered

Questionnaire of the study

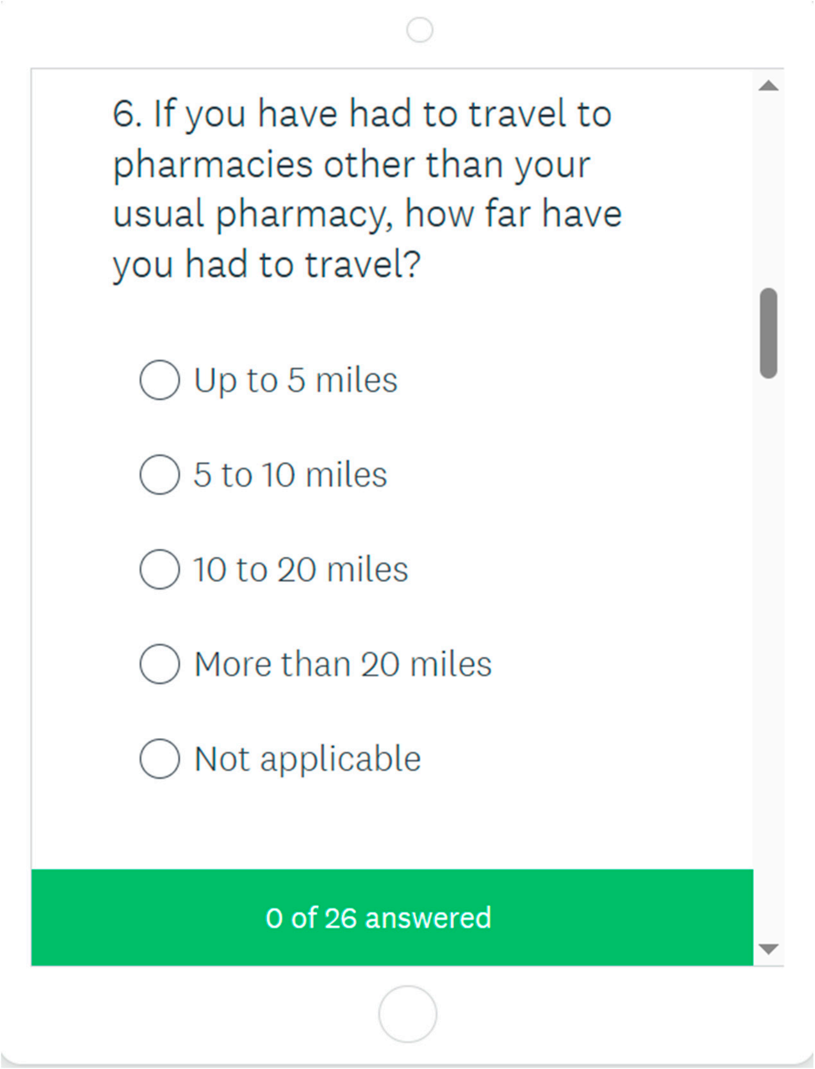

6. If you have had to travel to pharmacies other than your usual pharmacy, how far have you had to travel?

- ◯ Up to 5 miles
- ◯ 5 to 10 miles
- ◯ 10 to 20 miles
- ◯ More than 20 miles
- ◯ Not applicable

0 of 26 answered

Questionnaire of the study

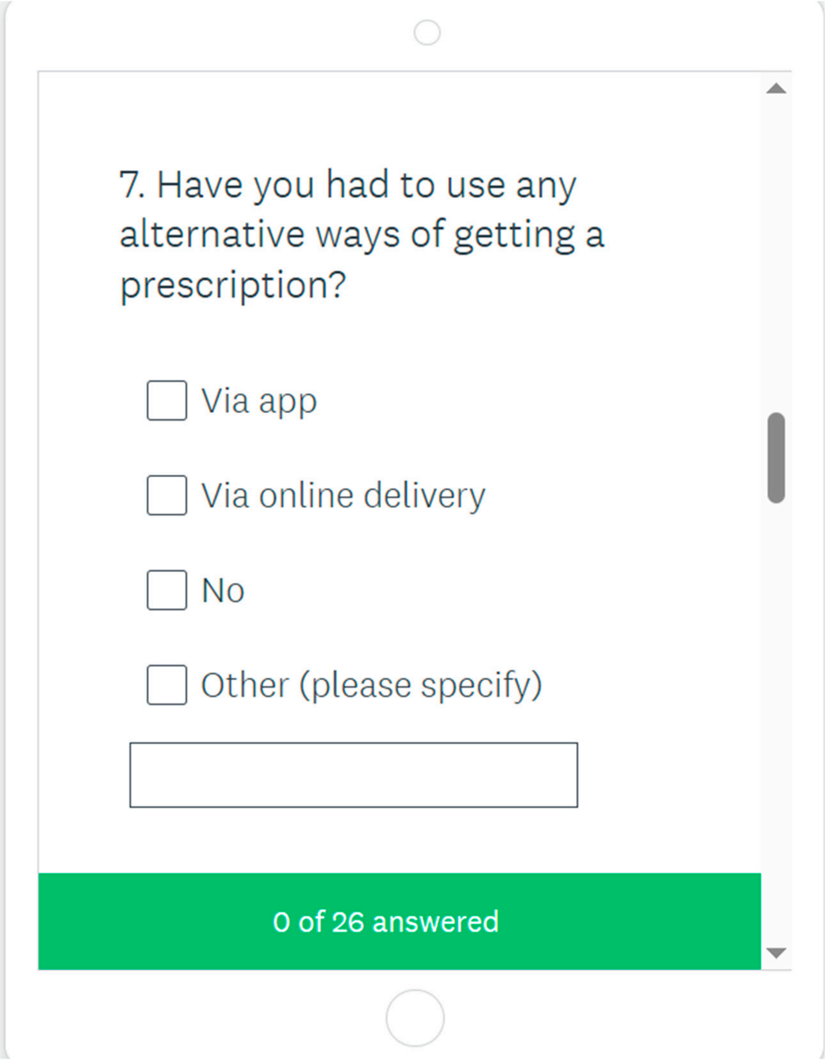

7. Have you had to use any alternative ways of getting a prescription?

- [ ] Via app
- [ ] Via online delivery
- [ ] No
- [ ] Other (please specify)

0 of 26 answered

Questionnaire of the study

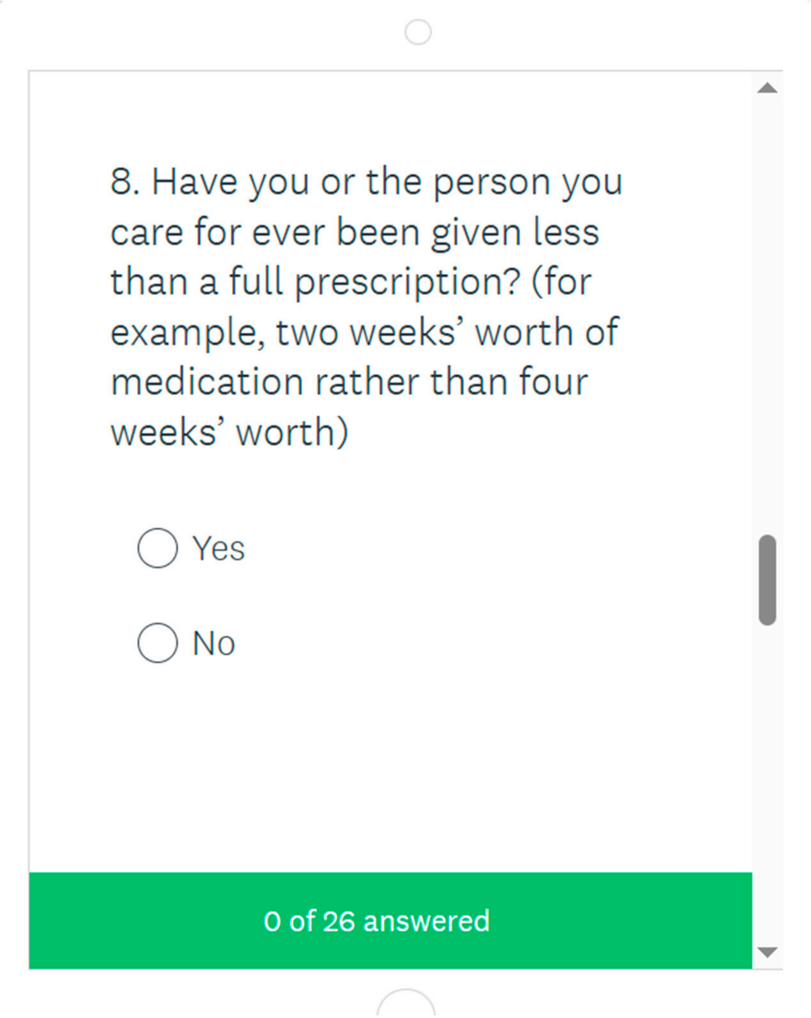

8. Have you or the person you care for ever been given less than a full prescription? (for example, two weeks' worth of medication rather than four weeks' worth)

- Yes
- No

0 of 26 answered

Questionnaire of the study

9. Have you or the person you care for experienced stress and/or anxiety as a result of a shortage of medication?

- ◯ Yes
- ◯ No

0 of 26 answered

Questionnaire of the study

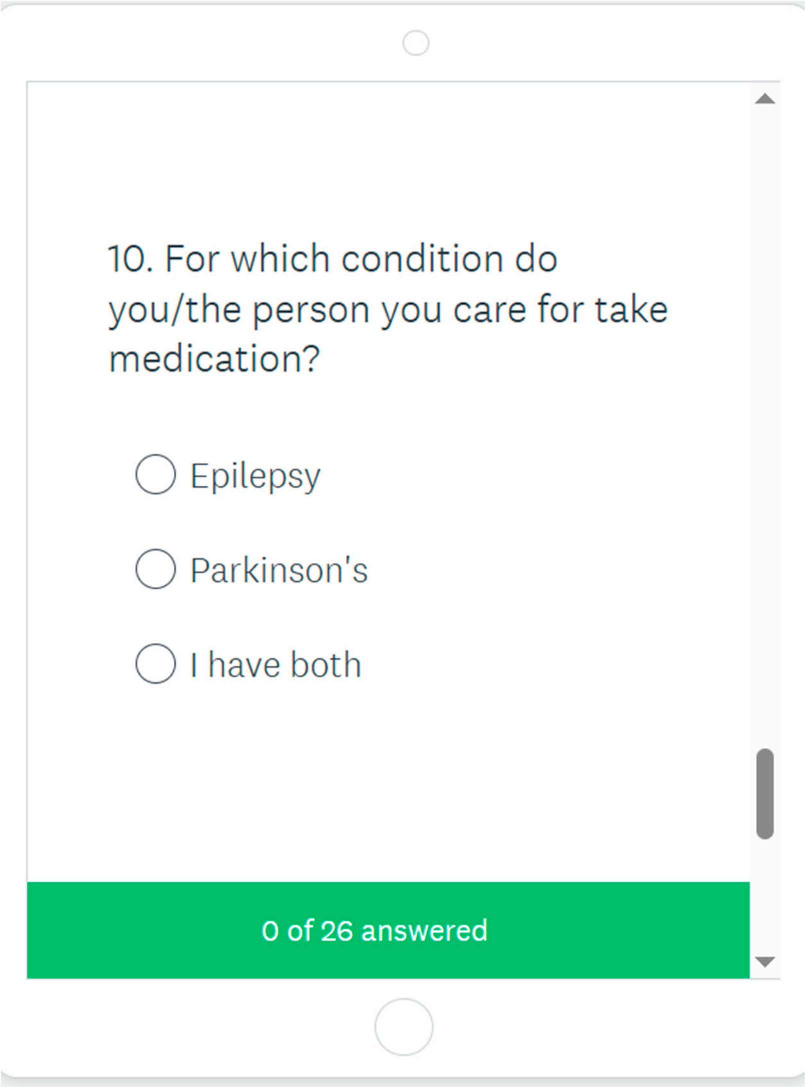

Questionnaire of the study

11. Have you or the person you care for ever been offered a different version or brand of epilepsy medication?

- ◯ Yes
- ◯ No

0 of 26 answered

Questionnaire of the study

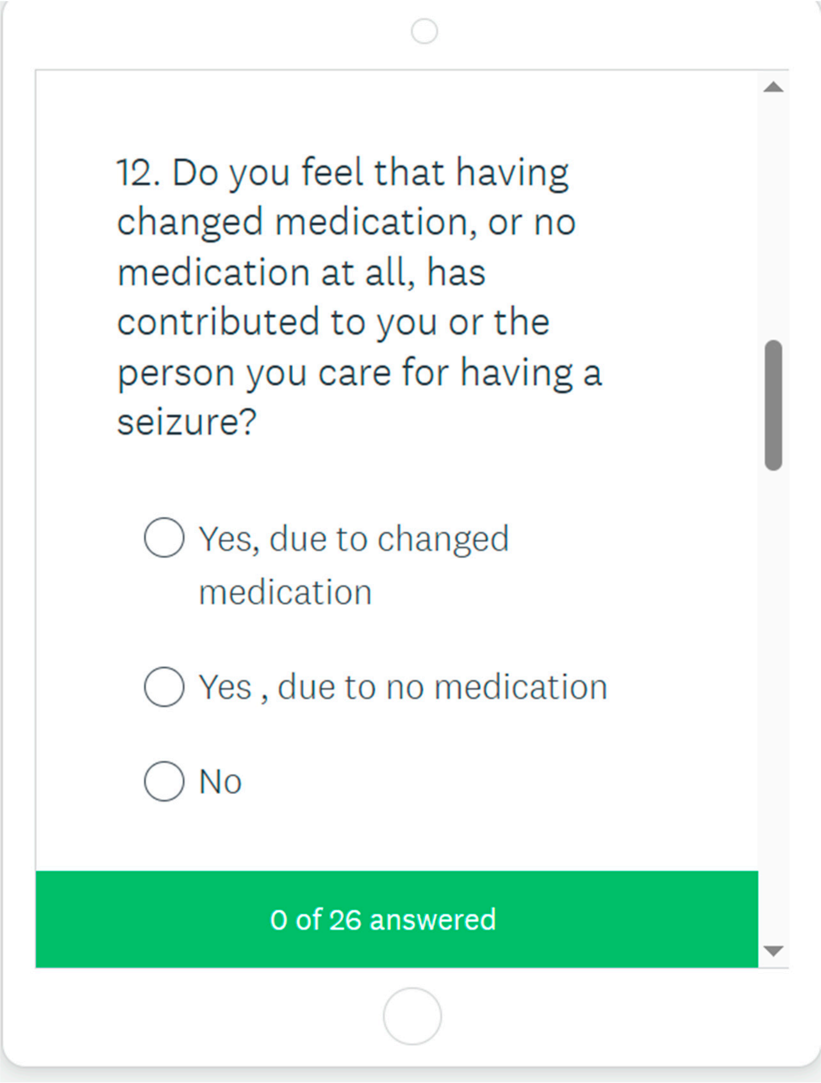

12. Do you feel that having changed medication, or no medication at all, has contributed to you or the person you care for having a seizure?

- Yes, due to changed medication
- Yes , due to no medication
- No

0 of 26 answered

Questionnaire of the study

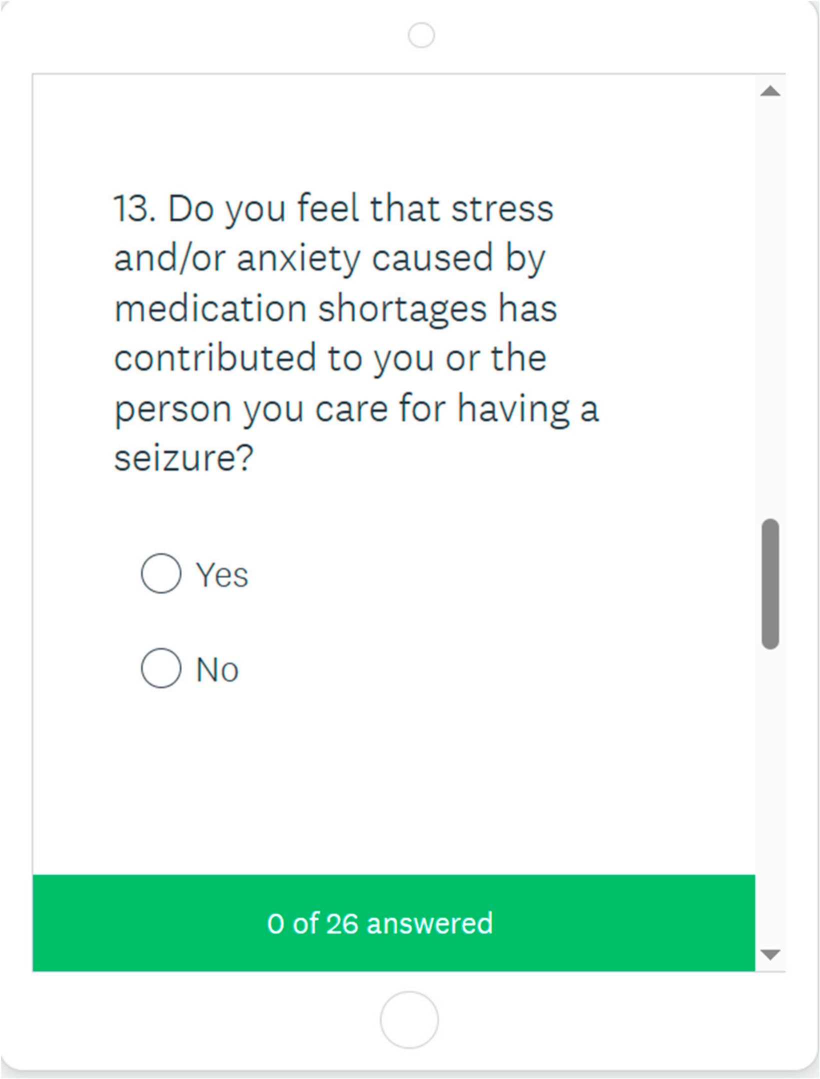
13. Do you feel that stress and/or anxiety caused by medication shortages has contributed to you or the person you care for having a seizure?

- Yes
- No

0 of 26 answered

Questionnaire of the study

14. Have you had a review of your epilepsy treatment in the last 12 months?

- ◯ Yes
- ◯ No

0 of 26 answered

Questionnaire of the study

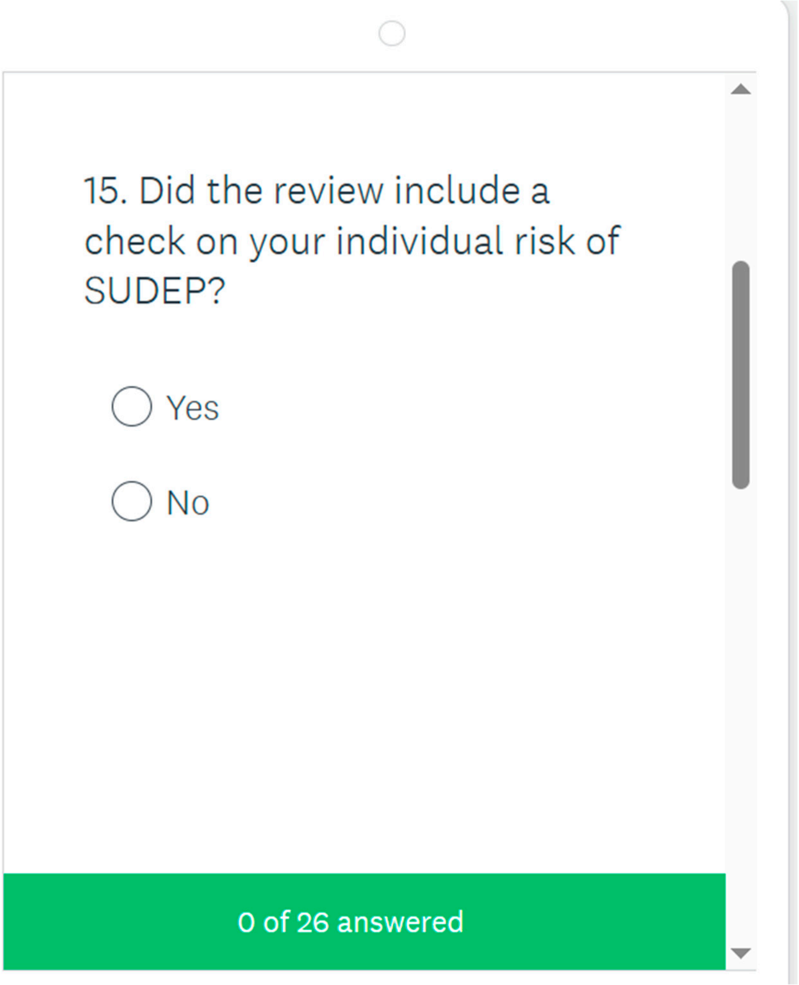
15. Did the review include a check on your individual risk of SUDEP?

- Yes
- No

0 of 26 answered

Questionnaire of the study

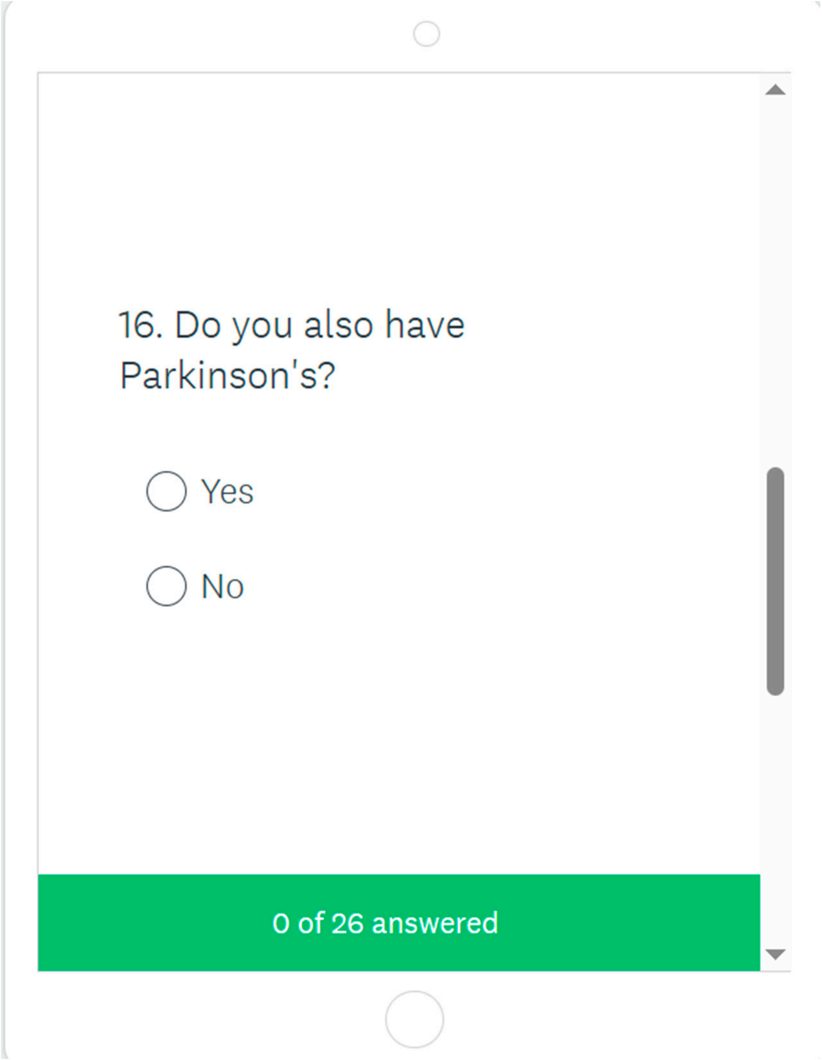
16. Do you also have Parkinson's?

- ◯ Yes
- ◯ No

0 of 26 answered

Questionnaire of the study

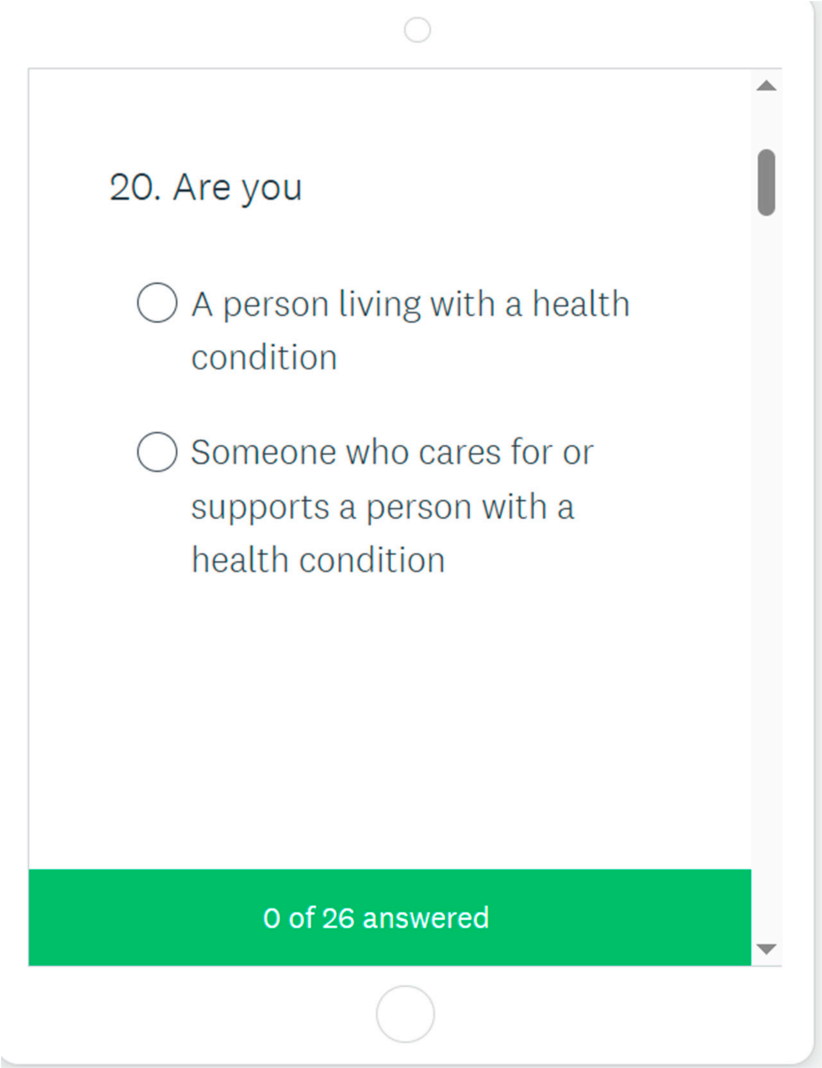

Questionnaire of the study

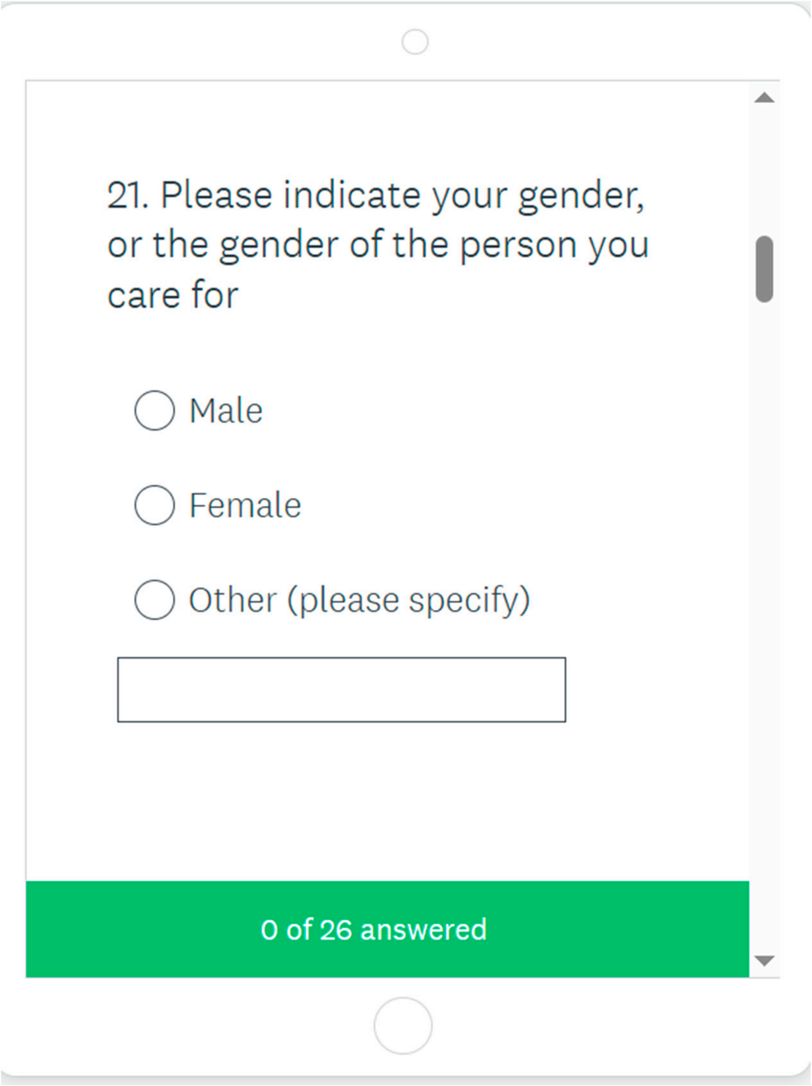
21. Please indicate your gender, or the gender of the person you care for

- ◯ Male
- ◯ Female
- ◯ Other (please specify)

0 of 26 answered

Questionnaire of the study

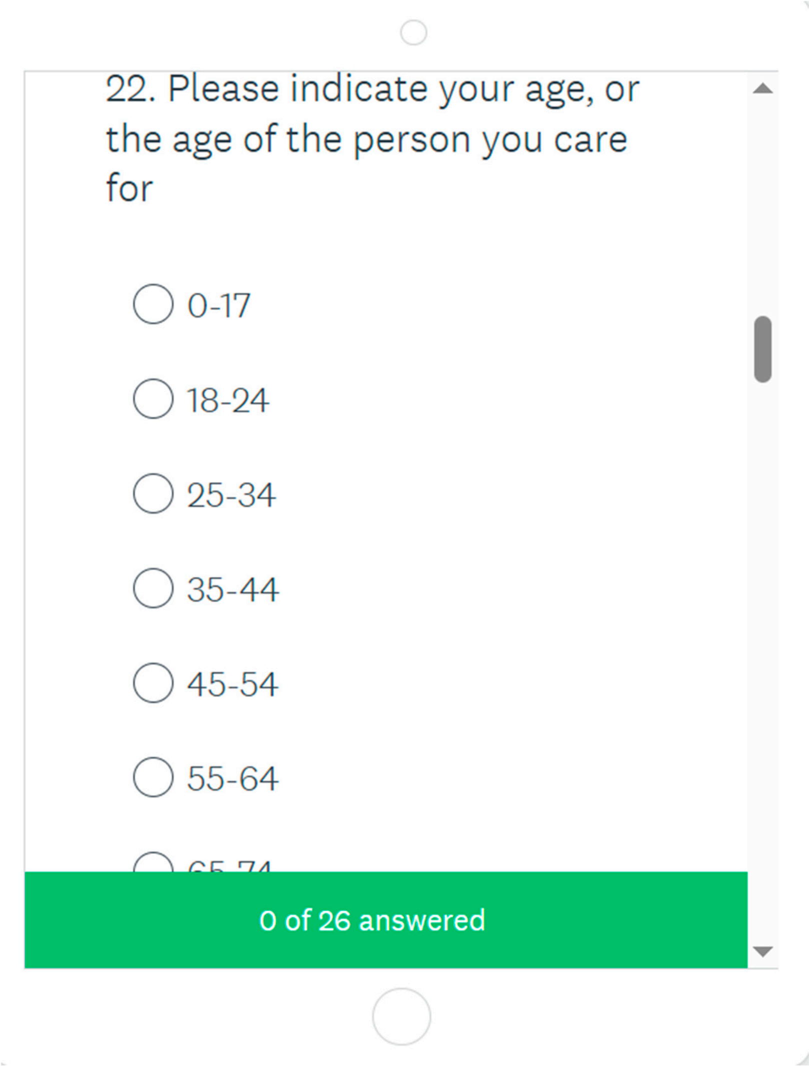
22. Please indicate your age, or the age of the person you care for

- 0-17
- 18-24
- 25-34
- 35-44
- 45-54
- 55-64
- [illegible]

0 of 26 answered

Questionnaire of the study

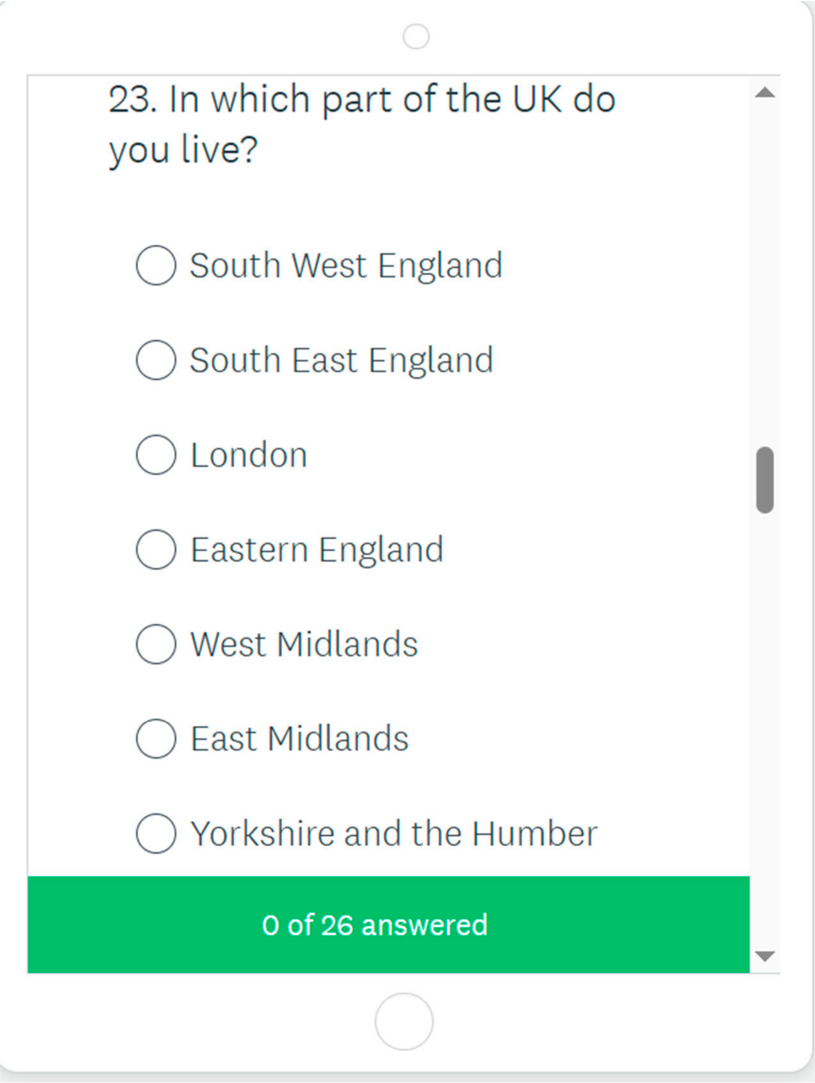
23. In which part of the UK do you live?

- South West England
- South East England
- London
- Eastern England
- West Midlands
- East Midlands
- Yorkshire and the Humber

0 of 26 answered
